# Mosaic-variegated aneuploidy syndrome mutation or haploinsufficiency in *Cep57* impairs tumor suppression

**DOI:** 10.1172/JCI120316

**Published:** 2018-07-23

**Authors:** Khaled Aziz, Cynthia J. Sieben, Karthik B. Jeganathan, Masakazu Hamada, Brian A. Davies, Raul O. Fierro Velasco, Nazneen Rahman, David J. Katzmann, Jan M. van Deursen

**Affiliations:** 1 Department of Biochemistry and Molecular Biology and; 2 Department of Pediatric and Adolescent Medicine, Mayo Clinic, Rochester, Minnesota, USA.; 3 Division of Genetics and Epidemiology, The Institute of Cancer Research, London, United Kingdom.

**Keywords:** Cell Biology, Oncology, Bone development, Genetic diseases

## Abstract

A homozygous truncating frameshift mutation in *CEP57 (CEP57^T/T^)* has been identified in a subset of mosaic-variegated aneuploidy (MVA) patients; however, the physiological roles of the centrosome-associated protein CEP57 that contribute to disease are unknown. To investigate these, we have generated a mouse model mimicking this disease mutation. *Cep57^T/T^* mice died within 24 hours after birth with short, curly tails and severely impaired vertebral ossification. Osteoblasts in lumbosacral vertebrae of *Cep57^T/T^* mice were deficient for Fgf2, a Cep57 binding partner implicated in diverse biological processes, including bone formation. Furthermore, a broad spectrum of tissues of *Cep57^T/T^* mice had severe aneuploidy at birth, consistent with the MVA patient phenotype. *Cep57^T/T^* mouse embryonic fibroblasts and patient-derived skin fibroblasts failed to undergo centrosome maturation in G_2_ phase, causing premature centriole disjunction, centrosome amplification, aberrant spindle formation, and high rates of chromosome missegregation. Mice heterozygous for the truncating frameshift mutation or a *Cep57*-null allele were overtly indistinguishable from WT mice despite reduced Cep57 protein levels, yet prone to aneuploidization and cancer, with tumors lacking evidence for loss of heterozygosity. This study identifies Cep57 as a haploinsufficient tumor suppressor with biologically diverse roles in centrosome maturation and Fgf2-mediated bone formation.

## Introduction

Rare hereditary human syndromes characterized by extensive genetic and phenotypic heterogeneity are among the most difficult diseases to understand at a molecular mechanistic level, and research on such syndromes relies heavily on mimicking the underlying mutations in animal models (1–5). One such example is mosaic-variegated aneuploidy (MVA) syndrome, in which biallelic mutations in *BUB1B* (encoding the protein BUBR1) (1), *CEP57* (6), or *TRIP13* (3) have been described. MVA presents with complex phenotypes that can include growth retardation, skeletal anomalies, facial dysmorphisms, microcephaly, heart defects, Dandy-Walker complex, seizures, hypothyroidism, ocular defects, and childhood cancers (3, 6, 7). Despite its genetic and phenotypic heterogeneity, a universal hallmark of MVA syndrome is the development of aneuploidy, an abnormal number of whole chromosomes, in a broad spectrum of tissues and organs throughout the body.

Mouse models mimicking MVA patients with alterations in BubR1 have provided important insights regarding cancer, aging, and age-related diseases (8, 9). Many missense mutations in these patients reduce protein stability, resulting in deficient BUBR1 levels (1). BUBR1 is a core component of the mitotic checkpoint, a surveillance mechanism that ensures proper attachment of metaphase spindles to kinetochores before sister chromatids segregate toward opposing spindles. A mouse model mimicking this common feature of *BUBR1* MVA patients is the BubR1 hypomorphic (*BubR1^H/H^*) model, which expresses approximately 10% of normal protein (8). *BubR1^H/H^* mice age prematurely, showing increased incidence of kyphosis, cataracts, and other progeroid phenotypes observed in MVA syndrome, such as growth retardation, muscle wasting, cardiac defects, and facial dysmorphisms (8, 10). Detailed characterization of multiple phenotypes in this model has contributed to discoveries in several biological processes. In arteries, BubR1 insufficiency reduced elasticity and deregulated responses that control vascular tone, both of which are features of vascular aging (11). In the brain, low BubR1 caused cerebral gliosis and reduced neurogenesis, a common feature of neurological aging (12). Furthermore, *BubR1* insufficiency predisposed the mice to carcinogen-induced tumorigenesis, a feature that recapitulates a key aspect of the human syndrome (13). The majority of the age-related pathologies observed in *BubR1^H/H^* mice are driven by cellular senescence (10), a discovery that helped establish a long-sought causal link between senescent cells and aging.

TRIP13 is an ATPase that converts the mitotic checkpoint protein MAD2 from a closed (active) to an open (inactive) conformation (14). In prometaphase, TRIP13 helps sustain mitotic checkpoint signaling. It does so by ensuring that a sufficiently large pool of open MAD2 is available in the mitotic cytosol for conversion to closed MAD2 by kinetochores that are not yet attached to spindle microtubules (15). In metaphase, when all kinetochores are attached, TRIP13 inactivates MAD2, thereby silencing the mitotic checkpoint and triggering anaphase onset (16). Like BUBR1 deficiency, TRIP13 deficiency impairs the mitotic checkpoint and causes chromosome missegregation and aneuploidy (3). TRIP13 mutations found in MVA patients have not yet been modeled in mice.

*CEP57* is perhaps the least studied of the MVA-associated genes, and how *CEP57* mutations in MVA patients promote aneuploidy is unknown. CEP57 (or translokin) was originally identified as a microtubule-binding protein that mediates nuclear translocation of FGF2 in aortic endothelial cells (17) and cancer cell lines (18). FGF2 is a pleiotropic growth factor involved in embryonic development, wound healing, angiogenesis, and tumor progression, but whether CEP57 is important for any of these biological functions is unclear (17). Subsequent work showed that CEP57 is a pericentriolar material (PCM) component that forms a complex with CEP152 and CEP63, two proteins that recruit the master regulator of centrosome duplication, PLK4, to centrosomes (19–23). Both depletion and overexpression of CEP57 result in centrosome amplification, although the role of PLK4 in this phenotype remains unclear (24, 25). Interestingly, mutations in *CEP63*, *CEP152*, and *PLK4*, but not *CEP57*, are associated with Seckel syndrome or autosomal recessive primary microcephaly syndrome, indicating that *CEP57* and its binding partners have nonredundant functions (2, 4, 5).

Consistent with the functions of several other PCM proteins, CEP57 is important for nucleation of microtubules at centrosomes (26). In preparation for microtubule nucleation in the context of mitotic spindle formation, many PCM components become enriched at centrosomes during G_2_ phase and early mitosis. This maturation process is driven by kinases, including PLK1 (27, 28). Beyond microtubule nucleation, PCM proteins regulate various other centrosomal processes, including centriole cohesion, engagement, and duplication (29). For example, CDK5RAP2 and pericentrin (PCNT) are important for centriole engagement (30–32), with cleavage of PCNT by separase disengaging centriole pairs during late mitosis (32). Centriole disengagement licenses the centriole for duplication in the following cell cycle (33), and the timing of this process is critical for proper centrosome duplication. Centrosome amplification is a common feature of cancer (34). Cells with extra centrosomes form multipolar mitotic spindles, generating daughter cells with highly abnormal karyotypes and reduced viability (35). Cells can, however, cluster amplified centrosomes to pseudo-bipolar spindles (36, 37). Often these spindles produce merotelic attachments (one kinetochore attached to multiple spindle poles) that can give rise to aneuploid karyotypes, thereby perpetuating chromosomal instability. The recent observation that transgenic mice with high Plk4 levels are tumor prone provides compelling evidence for the idea that centrosome amplification can lead to neoplastic transformation (38).

To better understand the biological consequences of *CEP57* mutations found in MVA syndrome, we generated a mouse model mimicking the most commonly mutated *CEP57* disease allele, c.915_925dup11 (6), as well as a *CEP57*-null allele. Whereas *Cep57^–/–^* mice died as embryos, mice homozygous for this disease mutation (*Cep57^T/T^*) died shortly after birth with defective vertebral ossification characterized by deficient Fgf2, a Cep57 binding partner that has been implicated in bone formation. We find that newborn *Cep57^T/T^* mice have severe aneuploidies in a broad spectrum of tissues. Mouse embryonic fibroblasts (MEFs) from these mice as well as skin fibroblasts from an MVA patient with the corresponding mutation failed to undergo centrosome maturation in G_2_ phase, causing premature centriole disjunction, centrosome amplification, aberrant spindle formation, and high rates of chromosome missegregation. *Cep57^+/T^* and *Cep57^+/–^* mice, on the other hand, had milder chromosomal instability phenotypes and were cancer prone. The latter finding was unexpected because *CEP57* MVA patients, unlike their *BUBR1* and *TRIP13* counterparts, have yet to be diagnosed with childhood cancers (3). Collectively, these results demonstrate that *Cep57^T^* is a partially functional allele, and that Cep57 is a multifunctional protein with important roles in centrosome maturation, tumor suppression, and Fgf2-mediated skeletal development.

## Results

### Cep57 controls Ffg2-mediated bone development.

**Figure 1:**
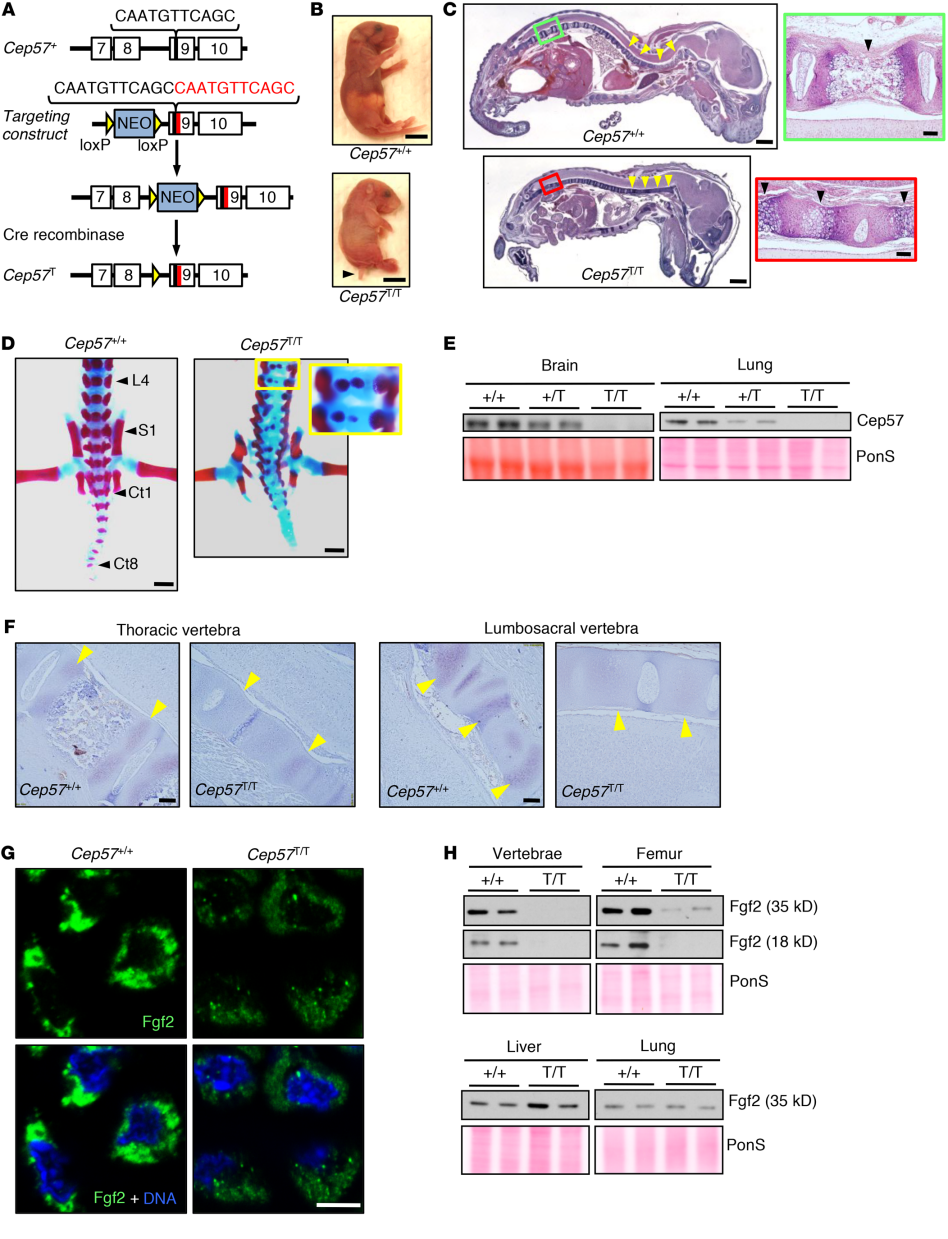
Cep57 controls Fgf2-mediated bone development. (**A**) Gene targeting approach used to mimic the *CEP57^T^* patient mutation. Shown are the relevant portion of the murine *Cep57* locus (top), the targeting vector (the 11-bp duplication is highlighted in red) with the recombined hypomorphic allele (middle), and the final *Cep57^T^* allele following Cre-mediated excision of the neomycin (NEO) selection cassette. (**B**) Images of pups several hours after birth (arrowhead marks the short curly tail). Scale bars: 5 mm. (**C**) H&E-stained sagittal sections of 1-day-old pups. Arrowheads indicate the degree of spinal cord curvature. Scale bars: 1 mm. High magnification of the areas marked by red and green boxes are shown to the right. Scale bars: 100 μm. (**D**) Images of alizarin red– and Alcian blue–stained bone (red) and cartilage (blue) tissue of 1-day-old pups. Indicated are lumbar vertebra 4 (L4), sacral vertebra 1 (S1), and caudal vertebra 1 and 8 (Ct1 and Ct8) landmarks. Inset (yellow box) shows defective (bifid) vertebral body ossification. Scale bars: 1 mm. (**E**) Western blot analysis of brain and lung lysates of 1-day-old *Cep57^+/+^*, *Cep57^+/T^*, and *Cep57^T/T^* mice. Ponceau (PonS) served as loading control. (**F**) Representative images of sagittal sections from thoracic and lumbosacral vertebral regions of 1-day-old pups immunostained for Fgf2. Arrowheads indicate regions with Fgf2 expression. Scale bars: 100 μm. (**G**) Analysis of Fgf2 subcellular localization using immunofluorescence in paraffin sections of the lumbosacral region of 1-day-old pups labeled for Fgf2. Nuclei were visualized with Hoechst. Scale bar: 5 μm. (**H**) Western blots of tissue lysates probed for Fgf2. Shown are the 35-kDa (full-length) and 18-kDa isoforms of Fgf2. PonS served as the loading control. Western blots are representative of 3 independent experiments.

A TALEN-mediated knock-in strategy was used to generate a mouse model for the *CEP57* c.915_925dup11 duplication mutation (hereafter referred to as *Cep57^T^*) that is frequently found in *CEP57* MVA syndrome patients and truncates the protein (ref. 6, Figure 1A, and Supplemental Figure 1A; supplemental material available online with this article; https://doi.org/10.1172/JCI120316DS1). Heterozygous mutant mice were obtained from 2 independently targeted embryonic stem cell clones and appeared overtly normal. *Cep57^T/T^* mice were live born albeit at below the expected Mendelian frequency (9% instead of 25%), indicating that a subset of homozygous mice died in utero (Supplemental Figure 1B). With 100% penetrance, newborn *Cep57^T/T^* mice had short curly tails, failed to feed, died within 24 hours, and were smaller than both *Cep57^+/T^* and WT littermates (Figure 1B). All *Cep57^T/T^* tissues and organs had a normal appearance upon routine histological evaluation with the exception of the vertebral column, which had limited curvature and lacked ossification in caudal vertebrae. The vertebral bodies in *Cep57^T/T^* pups were underdeveloped and lacked defined borders (Figure 1C). *Cep57^T/T^* pups stained with alizarin red and Alcian blue confirmed vertebral hypoplasia, incomplete bifid vertebral body ossification, and splayed pedicles (Figure 1D and Supplemental Figure 1C). All caudal vertebrae lacked ossification.

Neither WT Cep57 protein nor the expected truncated 40-kDa Cep57 protein was detectable on Western blots containing lysates of various tissues from newborn *Cep57^T/T^* mice. *Cep57^+/T^* tissue lysates showed a reduction in Cep57 compared with corresponding tissues of *Cep57^+/+^* mice (Figure 1E). These findings raised the possibility that *Cep57^T/T^* mice were virtual *Cep57* knockouts. To test this idea, we generated a *Cep57* knockout allele by deleting exons 1–4 using CRISPR/Cas9 technology in fertilized eggs (Supplemental Figure 1D). Interbreeding of *Cep57^+/–^* mice yielded viable E13.5 *Cep57^–/–^* embryos; however, no live *Cep57^–/–^* embryos were detected at E15.5 (Supplemental Figure 1B). The observation that *Cep57^–/–^* mice die much earlier than *Cep57^T/T^* mice indicates that the *Cep57*^T^ allele is expressed at low levels and that the resulting truncated protein has some residual biological activity.

To understand the molecular basis for the defects in vertebral bone development of *Cep57^T/T^* mice, we focused on Fgf2 for 2 reasons. First, Fgf2 has been shown to stimulate bone formation and is expressed in osteoblastic cells (39, 40). Second, Fgf2 is an established binding partner of Cep57 involved in Fgf2 nuclear translocation (17). Immunohistochemistry (IHC) on sagittal paraffin sections of 1-day-old WT pups revealed that Fgf2 is abundant in bone tissue, including all vertebrae (Figure 1F). Within vertebrae, Fgf2 staining was restricted to osteoblast-rich regions. In contrast, Fgf2 levels in bone were consistently lower in *Cep57^T/T^* mice of the same age, with lumbar, sacral, and caudal vertebrae showing little or no Fgf2 staining. We complemented the immunohistochemical stainings with immunofluorescence to examine whether the subcellular localization of Fgf2 was altered in *Cep57^T/T^* mice. We found that Fgf2 in bone tissue was localized in the cytoplasmic compartment irrespective of *Cep57* status (Figure 1G). Consistent with our IHC data, Fgf2 levels by immunofluorescence were markedly lower in *Cep57^T/T^* mice. Western blot analysis confirmed that Fgf2 levels were reduced in vertebrae of *Cep57^T/T^* mice (Figure 1H). In addition, Fgf2 levels were also reduced in long bones such as the femur, albeit to a lesser extent than in vertebrae. We included liver and lung in our analysis to assess tissue selectivity of these reductions. In both these tissues, *Cep57^+/+^* and *Cep57^T/T^* mice contained similar amounts of Fgf2, suggesting that Fgf signaling defects are exclusive to bone in these mice (Figure 1H).

Fgf2 activates both MAPK/ERK and PI3K/Akt signaling (41, 42). IHC on sections from 1-day-old pups revealed that active phosphorylated Akt (p-Akt) was unchanged in distal vertebrae of *Cep57^T/T^* mice, whereas active ERK was reduced (Supplemental Figure 1E). Importantly, the decrease in Fgf2 abundance observed in *Cep57^T/T^* vertebrae had no transcriptional basis as evidenced by quantitative reverse transcriptase PCR (qRT-PCR) (Supplemental Figure 1F). Collectively, these data demonstrate that homozygosity for the c.915_925dup11 duplication mutation is not compatible with postnatal development in mice, and uncover a previously unknown role for Cep57 in bone development, presumably through its ability to interact with and regulate the abundance of Fgf2, a key modulator of ossification.

### Cep57 loss or truncation leads to supernumerary centrosomes.

**Figure 2:**
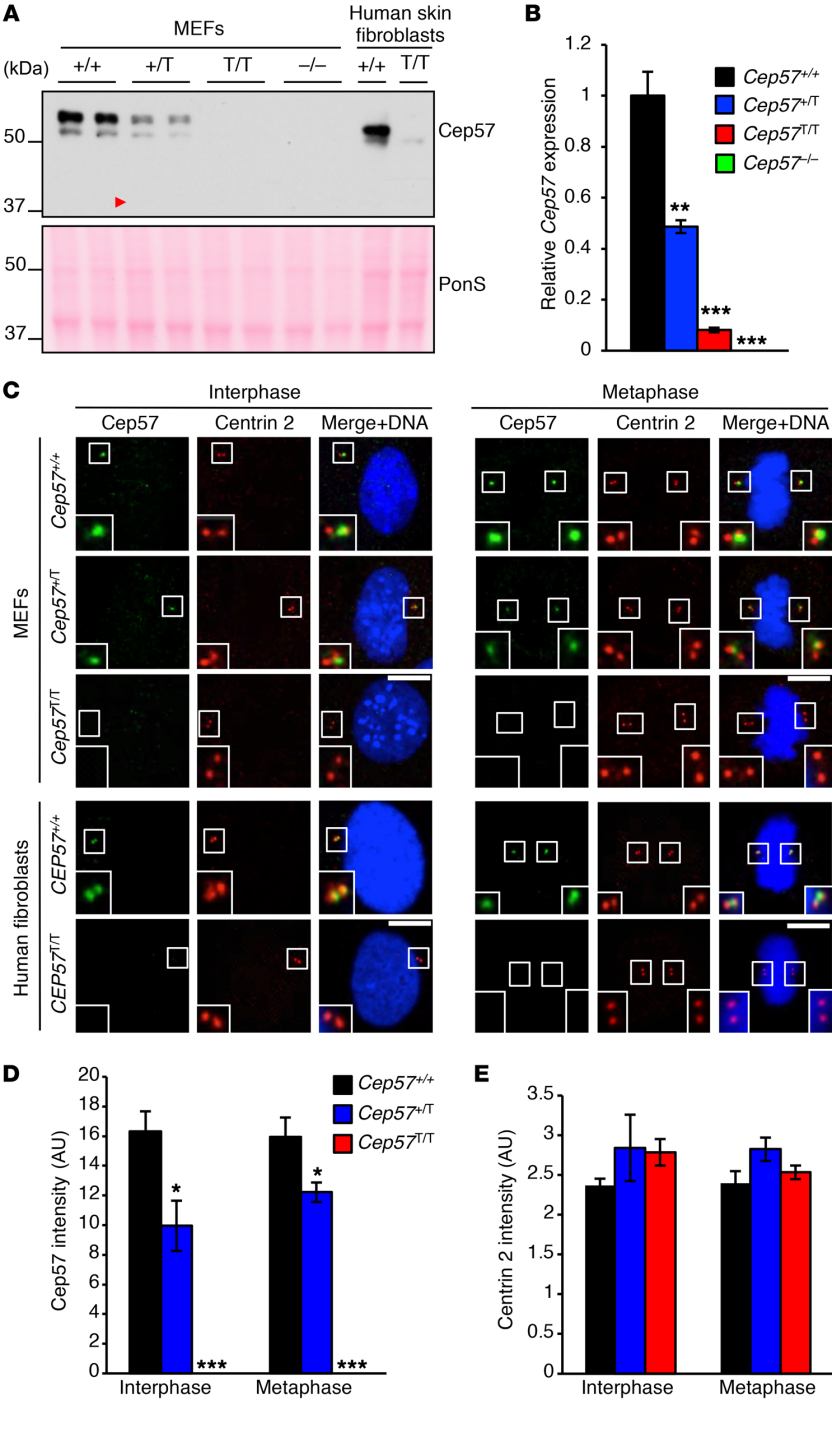
Cep57 is undetectable in *Cep57^T/T^* MEFs and *CEP57^T/T^* patient fibroblasts. (**A**) Western blots of MEF lysates comparing Cep57 expression levels among various genotypes as well as normal human fibroblasts and *CEP57^T/T^* MVA patient fibroblasts. The red arrowhead indicates the predicted size of the truncated Cep57^T^ product (40 kDa). Ponceau (PonS) served as loading control. (**B**) Quantitative reverse transcriptase PCR analysis of *Cep57* transcript expression in MEFs of indicated genotypes. Three independent lines were evaluated per genotype, and quantitative PCR run in triplicate. (**C**) Representative images of interphase and metaphase MEFs, and normal human and MVA patient fibroblasts labeled for Cep57 and centrin 2. Insets display magnified images of centrosomes. Scale bars: 5 μm. (**D**) Quantification of Cep57 intensity as seen in **C**. (**E**) Quantification of centrin 2 intensity as seen in **C**. Analyses in **B**, **D**, and **E** were performed on at least 3 independent lines per genotype (20 cells per line). Data represent the mean ± SEM. Western blots are representative of 3 independent experiments. Statistical significance in **B** and **E** was determined using 1-way ANOVA followed by Tukey’s multiple-comparisons test; statistical analysis in **D** was performed using a 2-tailed unpaired *t* test. *P < 0.05, **P < 0.01, ***P < 0.001.

MVA syndrome patients exhibit chromosome number instability throughout tissues and organs, irrespective of the underlying gene mutation. However, for Cep57, little is known about its biological function in chromosome segregation, and how this may be perturbed in MVA syndrome patients. To address these questions, we generated MEFs from embryonic day 13.5 (E13.5) *Cep57^T/T^*, *Cep57^+/T^*, *Cep57^+/+^*, *Cep57^+/–^*, and *Cep57^–/–^* embryos. The frequencies with which *Cep57^T/T^* and *Cep57^–/–^* embryos were obtained from heterozygous intercrosses were 19% and 15%, respectively (Supplemental Figure 1B), indicating that some of the embryos died before E13.5. No Cep57 was detected on Western blots containing *Cep57^T/T^* and *Cep57^–/–^* MEF lysates probed with antibodies raised against the N-terminus of Cep57 (Figure 2A). As expected, Cep57 levels were low in *Cep57^+/–^* MEFs (Supplemental Figure 2A). qRT-PCR analysis revealed that *Cep57^T/T^* MEFs contained very low amounts of *Cep57* transcript (~8%), whereas *Cep57^+/T^* MEFs had approximately 50% of normal *Cep57* transcripts compared with WT MEFs (Figure 2B). The lack of detectable levels of truncated Cep57 protein was further confirmed in skin fibroblasts derived from a patient carrying the identical homozygous *CEP57* mutation (hereafter referred to as *CEP57^T/T^* MVA fibroblasts; Figure 2A). Cep57 localized to centrosomes throughout the cell cycle in both *Cep57^+/+^* MEFs and normal human fibroblasts, as demonstrated by immunofluorescence using an antibody against the Cep57 N-terminus (Figure 2C and Supplemental Figure 2B). In contrast, no centrosome labeling was observed in *Cep57^T/T^* MEFs and *CEP57^T/T^* MVA fibroblasts, whereas Cep57 signals in *Cep57^+/T^* MEFs were overtly reduced compared with their WT counterparts (Figure 2, C and D). Centriole size did not differ between the mutants and WT MEFs (Figure 2E).

**Figure 3:**
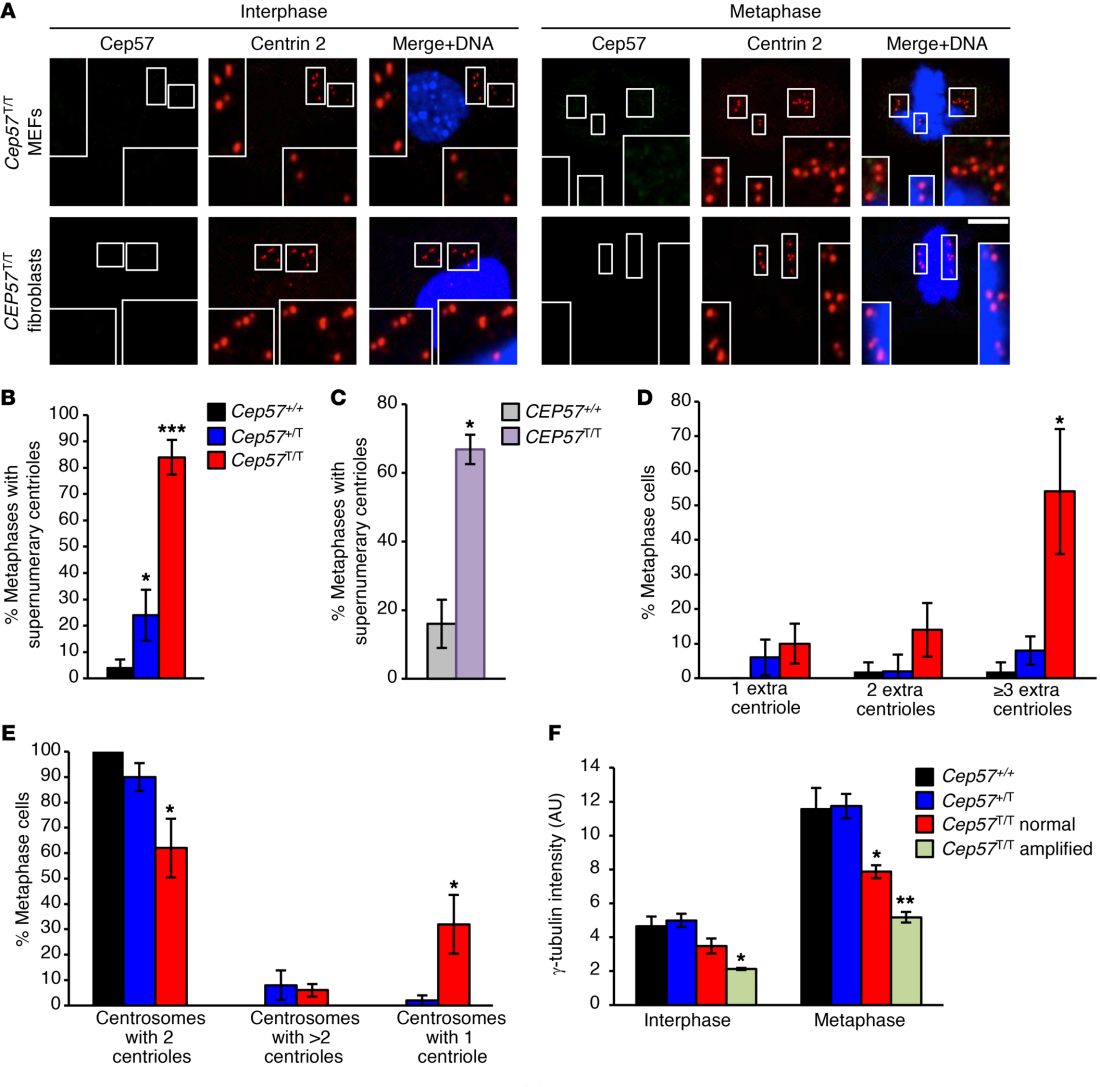
Cep57 loss or truncation leads to supernumerary centrosomes. (**A**) Representative images of interphase and metaphase MEFs and MVA patient fibroblasts with centrosome amplification labeled for Cep57 and centrin 2. Insets display magnified images of centrioles. Scale bar: 5 μm. (**B**) Quantification of the percentage of MEFs with centriole amplification. (**C**) Quantification of human fibroblasts of indicated genotypes with centriole amplification. (**D**) Subcategorization of cells with amplified centrioles by severity of amplification (number of extra centrioles per metaphase cell). (**E**) Quantification of number of centrioles (centrin 2) per centrosome (γ-tubulin foci). (**F**) Intensity of γ-tubulin quantified in MEFs. Analyses in **B** and **D**–**F** were performed on at least 5 independent lines per genotype (10 cells per line). Analysis in **C** was performed on 1 cell line. At least 25 cells were scored. Experiment was repeated 3 times. Data represent mean ± SEM. Statistical significance in **B** and **D**–**F** was determined using 1-way ANOVA followed by Tukey’s multiple-comparisons test; statistical analysis in **C** was performed using a 2-tailed unpaired *t* test. *P < 0.05, **P < 0.01, ***P < 0.001.

While performing these immunolocalization studies, we observed that a high proportion of *Cep57^T/T^* MEFs and *CEP57^T/T^* MVA fibroblasts had supernumerary centrioles (Figure 3A). To conduct an in-depth analysis of this phenotype, we colabeled centrin 2 and γ-tubulin, a key component of the pericentriolar material (PCM) important for microtubule nucleation, in *Cep57^T/T^*, *Cep57^+/T^*, and *Cep57^+/+^* MEFs (Supplemental Figure 2C). We found that the percentage of cells with supernumerary centrioles in metaphase was 84% and 24% in *Cep57^T/T^* and *Cep57^+/T^* MEFs, respectively, with just 4% of *Cep57^+/+^* MEFs showing this phenotype (Figure 3B). Similar results were obtained with *CEP57^T/T^* and *CEP57^+/+^* human fibroblasts (67% and 18%; Figure 3C). Fifty-four percent of *Cep57^T/T^* metaphase cells had 3 or more extra centrioles compared with 8% and 2% of *Cep57^+/T^* and *Cep57^+/+^* metaphase cells, respectively (Figure 3D). In addition, 38% of *Cep57^T/T^* metaphase cells had aberrant centrosomes that consisted of either 1 centriole (32%) or more than 2 centrioles (6%) (Figure 3E). *Cep57^+/+^* metaphase cells had no such defects compared with 10% of *Cep57^+/T^* metaphase cells. Strikingly, *Cep57^T/T^* metaphase centrosomes were less robustly labeled for γ-tubulin than *Cep57^+/T^* and *Cep57^+/+^* metaphase centrosomes, which was confirmed by measurements of signal intensities (Figure 3F). The decline was most profound in *Cep57^T/T^* metaphase cells containing extra centrosomes. In interphase cells the decline in γ-tubulin was less profound and was significantly altered only in *Cep57^T/T^* cells with amplified centrosomes.

### PCM maturation is Cep57 dependent.

**Figure 4:**
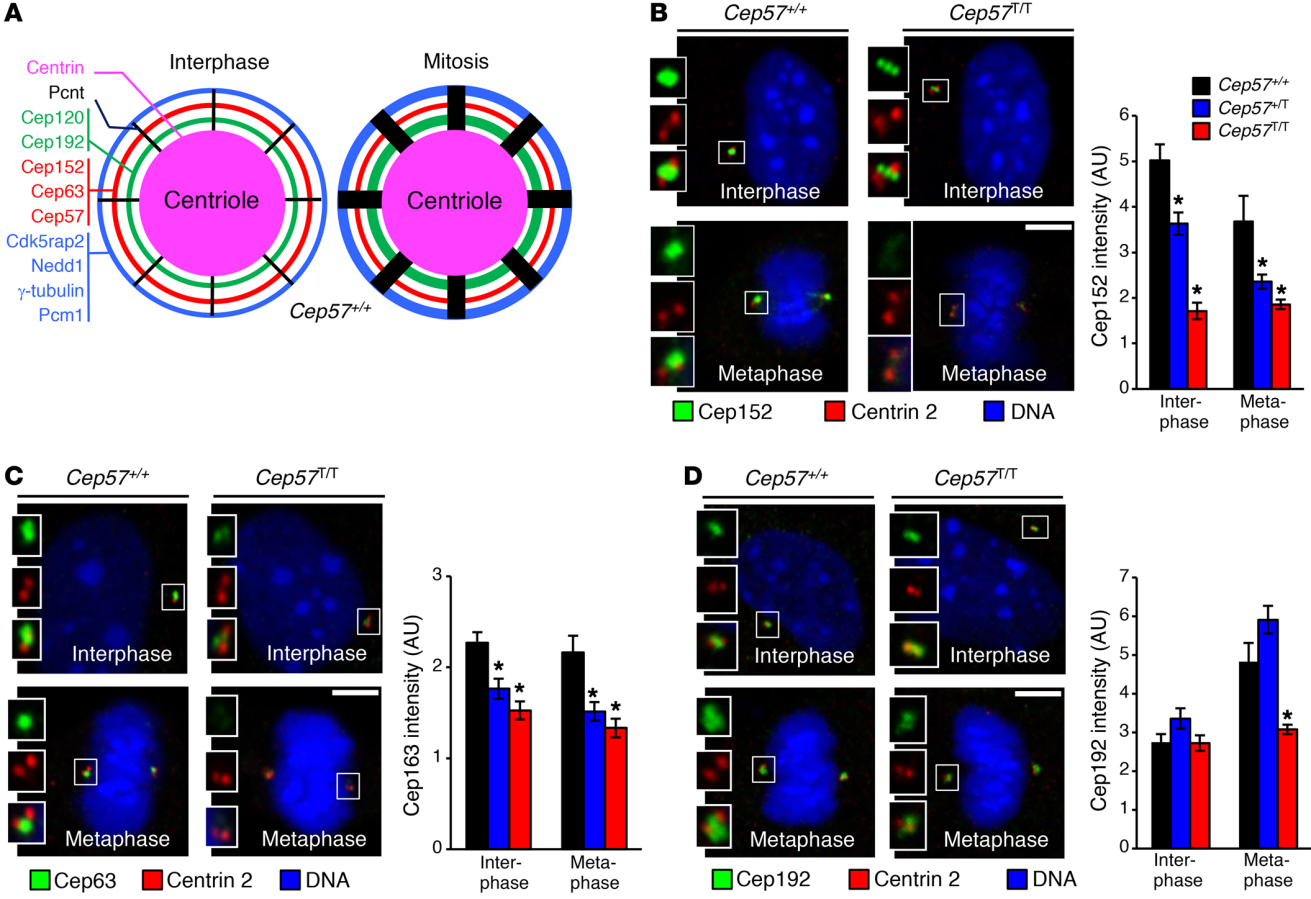
Cep57 truncation impairs recruitment of binding partners. (**A**) Schematic representation of PCM organization in interphase and mitotic WT cells. Thickness of rings correlates with the amount of PCM component. (**B**) Representative images from interphase and metaphase MEFs labeled for Cep152 and centrin 2. Quantification of Cep152 signal intensity is shown on the right. (**C**) Representative images from interphase and metaphase MEFs labeled for Cep63 and centrin 2. Quantification of Cep63 signal intensity is shown on the right. (**D**) Representative images from interphase and metaphase MEFs labeled for Cep192 and centrin 2. Quantification of Cep192 signal intensity is shown on the right. Data represent mean ± SEM. Statistical significance in **B**–**F** was determined using 1-way ANOVA followed by Tukey’s multiple-comparisons test. *P < 0.05, **P < 0.01, ***P < 0.001. Scale bars: 5 μm.

**Figure 5:**
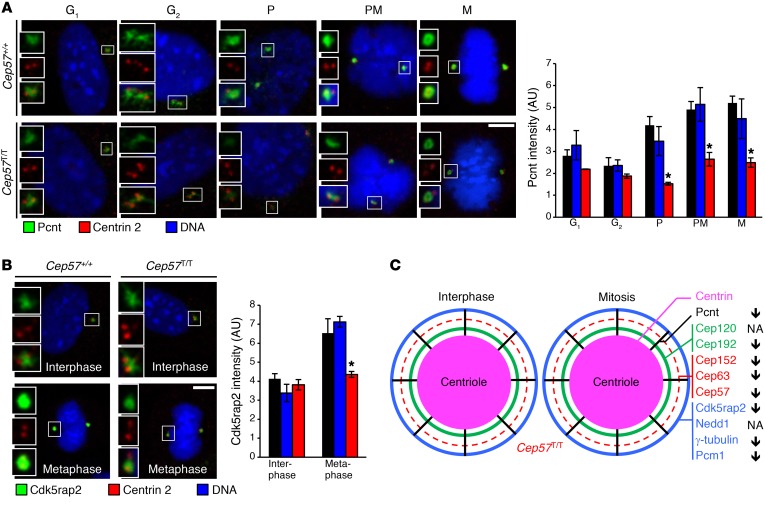
Cep57 truncation perturbs centrosome maturation. (**A**) Representative images from MEFs in G_1_, G_2_, prophase (P), prometaphase (PM), and metaphase (M) phases of the cell cycle labeled for pericentrin (PCNT) and centrin 2. Quantification of PCNT signal intensity is shown on the right. (**B**) Representative images from interphase and metaphase MEFs labeled for Cdk5rap2 and centrin 2. Quantification of Cdk5rap2 signal intensity is shown on the right. (**C**) Schematic representation of PCM organization in interphase and mitotic *Cep57^T/T^* cells. Thickness of rings correlates to the amount of PCM component. Arrows on the right indicate increase or decrease of PCM component in *Cep57^T/T^* cells compared with WT cells. Dashed line indicates reduction in accumulation of Cep57, Cep63, and Cep152. NA, not applicable. Data represent mean ± SEM. Statistical significance was determined using 1-way ANOVA followed by Tukey’s multiple-comparisons test. *P < 0.05, **P < 0.01, ***P < 0.001. Scale bars: 5 μm.

The observed changes in γ-tubulin levels in *Cep57^T/T^* MEFs prompted a more comprehensive analysis of the PCM surrounding the mother centriole in both interphase and mitosis. The PCM is organized into concentric toroid layers consisting of specific subsets of PCM proteins, and radial struts consisting of Pcnt (Figure 4A). The PCM proteins within these structures increase in abundance as PCM grows in size during the process of centrosome maturation late in G_2_ phase, with the exception of the Cep152-Cep63-Cep57 layer, which remains constant throughout the cell cycle (43–46). Levels of both Cep152 and Cep63 were reduced in *Cep57^T/T^* MEFs in both interphase and mitosis (Figure 4, B and C). Significant yet less profound reductions in both proteins were also observed in *Cep57^+/T^* MEFs, which have reduced Cep57 at centrosomes. Importantly, even though the Cep63-Cep152-Cep57 ring complex enables centrosomal accumulation of Plk4 (19–22), centrosomal recruitment of this regulator of centriole duplication was not altered in *Cep57^T/T^* MEFs (Supplemental Figure 3A). Immunolocalization experiments revealed that the association of PCM proteins representative of other concentric rings (Cep192 and Cdk5rap2) or the radial struts (Pcnt) was normal in *Cep57^T/T^* interphase cells, but was consistently reduced in mitosis (Figure 4D; Figure 5, A and B; and Supplemental Figure 3B). No such changes were observed in *Cep57^+/T^* MEFs. Collectively, these results identify Cep57 as a critical regulator of centrosome maturation (Figure 5C). Immunostaining of *CEP57^+/+^* and *CEP57^T/T^* MVA fibroblasts for CEP63, CEP152, PCNT, and CDK5RAP2 further strengthened this conclusion (Supplemental Figure 3, C–F).

### Cep57 controls proper timing of centriole disengagement in mitosis.

Besides Plk4 overexpression, centrosome amplification can be caused by cytokinesis failure or centrosome disjunction, or by premature centriole disengagement (34). Live-cell imaging of *Cep57^T/T^* and *Cep57^+/+^* MEFs expressing H2B-mRFP indicated that cytokinesis failure is not the source of the supernumerary centrosome phenotype in *Cep57^T/T^* MEFs (Supplemental Figure 4A). Consistent with this result, the proportion of cells with 4N DNA content was not increased in *Cep57^T/T^* MEFs compared with *Cep57^+/+^* MEFs (Supplemental Figure 4B). Failure of centrosome disjunction was also unlikely, as distances between duplicated centrosomes in G_2_ phase were similar for *Cep57^T/T^*, *Cep57^+/T^*, and *Cep57^+/+^* MEFs (Supplemental Figure 4C).

**Figure 6:**
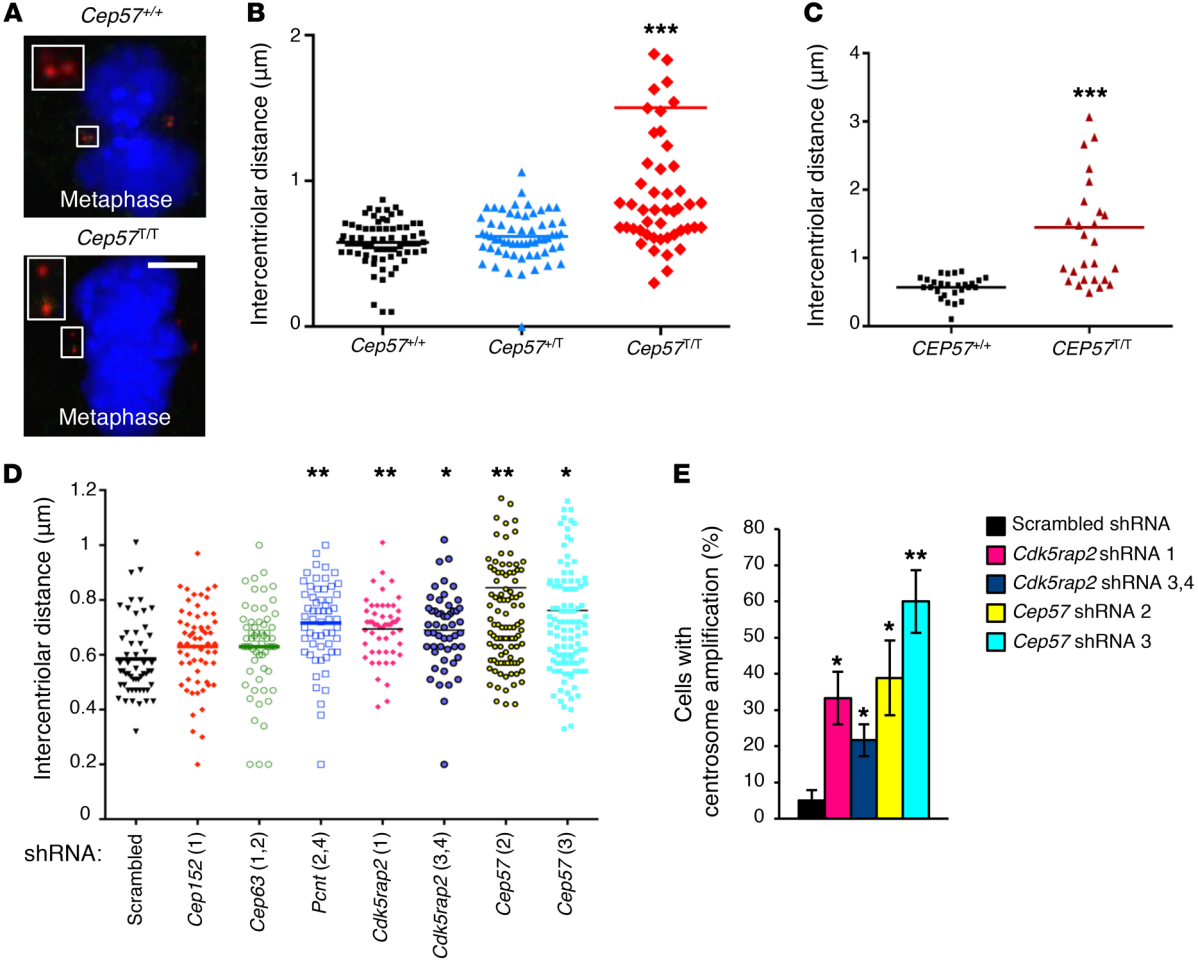
Cep57 controls the timing of centriole disengagement in mitosis. (**A**) Representative images of MEFs labeled for centrin 2 to measure intercentriolar distance. Scale bar: 5 μm. (**B**) Dot plot of metaphase intercentriolar distance measurements in MEFs of indicated genotypes. Twelve data points for *Cep57^T/T^* are outside the range shown. (**C**) Dot plot of metaphase intercentriolar distance measurements in human fibroblasts of indicated genotypes. One data point (*CEP57^T/T^*) is outside the range shown. (**D**) Dot plot of metaphase intercentriolar distance measurements in WT MEFs transduced with indicated shRNAs. (**E**) Quantification of the percentage of cells with centrosome amplification after knockdown using indicated shRNAs. Analyses in **B**, **D**, and **E** were performed on at least 3 independent lines per genotype (20 cells per line). Analysis in **C** was performed on 1 line per genotype (at least 20 cells per line). The experiment was repeated 3 times. Data in **E** represent the mean ± SEM. Statistical significance in **B** was determined using 1-way ANOVA followed by Tukey’s multiple-comparisons test. Statistical significance in **C**–**E** was determined using a 2-tailed unpaired *t* test. *P < 0.05, **P < 0.01, ***P < 0.001.

To screen for premature centriole disengagement, we labeled *Cep57^T/T^*, *Cep57^+/T^*, and *Cep57^+/+^* MEFs for centrin 2, selected metaphase cells that lacked centrosome amplification and had only 1 pair of centrioles on each side of the metaphase plate, and measured the intercentriolar distance of paired centrioles (47). We used a distance greater than 1 μm as a measure for premature centriole disengagement. The distance between paired centrioles was virtually always less than 1 μm in *Cep57^+/T^* and *Cep57^+/+^* metaphase cells, which is consistent with full centriole engagement (Figure 6, A and B). In contrast, a significant proportion of centrosomes in *Cep57^T/T^* MEFs had intercentriolar distances exceeding 1 μm, indicative of precocious disengagement. No premature disengagement was observed in *Cep57*-insufficient MEFs in G_2_ phase, indicating that the defect is occurring in mitosis (Supplemental Figure 4, C and D). Likewise, a considerable proportion of *CEP57^T/T^* MVA patient fibroblasts showed evidence of early centriole disengagement in metaphase (Figure 6C). We validated that the above measurements accurately represent mother-daughter intercentriolar distances by immunolabeling cells for the daughter centriole marker cenexin and the mother centriole appendage marker centrobin (Supplemental Figure 4, E and F).

An independent assay for centriole disengagement involves c-Nap1 and centrin 2 coimmunolabeling, with a c-Nap1/centrin 2 intensity ratio of 1:2 representing centriole engagement and a 1:1 ratio representing disengagement (47). We were only successful in applying this assay on *CEP57^T/T^* MVA patient fibroblasts, because MEFs did not reliably exhibit c-Nap1 labeling in metaphase. Consistent with our results from intercentriolar distance measurements, we found premature centriole disengagement in a significant proportion of *CEP57^T/T^* MVA patient fibroblasts in metaphase (Supplemental Figure 4G). Given that premature disengagement licenses centrioles for unwarranted centriole duplication and causes supernumerary centrosomes (33), it is very plausible that the centrosome amplification phenotype observed in *Cep57^T/T^* MEFs and *CEP57^T/T^* MVA patient fibroblasts is a direct consequence of premature centriole disengagement in metaphase. We further note that measurements of c-Nap1/centrin 2 intensity at centrosomes of interphase cells revealed a ratio of 1:1 in virtually all cells, irrespective of CEP57 status, confirming that premature disengagement in Cep57-deficient cells occurs in mitosis (Supplemental Figure 4, H and I).

Cleavage of Pcnt by separase drives centriole disengagement (31, 32), raising the possibility that the decrease in centrosome-associated Pcnt observed in *Cep57^T/T^* MEFs underlies their precocious disengagement phenotype. Furthermore, mouse models mimicking Cdk5rap2 mutations found in Seckel syndrome are also characterized by low centrosomal Pcnt levels and premature disengagement (30). To validate that decreases in centrosome-associated Pcnt and Cdk5rap2 cause precocious centriole disengagement, we knocked each of these 2 proteins down in *Cep57^+/+^* MEFs using lentivirally expressed shRNAs (Supplemental Figure 5, A–C) and measured the intercentriolar distance in metaphase. Indeed, both knockdowns yielded a significant increase in centriole separation (Figure 6D), indicative of premature centriole disengagement. In addition, knockdown of *Cdk5rap2* significantly increased the incidence of centrosome amplification (Figure 6E). Consistent with a reported cellular phenotype of *Pcnt^–/–^* MEFs (48), *Pcnt* knockdown resulted in centrosomes that often oriented non-perpendicularly to the metaphase plate but maintained numerical integrity, implying that centrosome amplification is dependent on Pcnt functions beyond its role in regulating the timing of centriole disengagement.

Depletion of Cep63 or Cep152 results in centriolar loss rather than amplification (22, 23). Consistent with these observations, we found that knockdown of *Cep63* or *Cep152* in *Cep57^+/+^* MEFs increased the incidence of mitotic centrosomes with only 1 centriole, and there was no evidence of centrosome amplification (Supplemental Figure 5, A, D, and E, and data not shown). Moreover, intercentriolar distances in metaphases that had centrosomes with 2 centrioles were normal (Figure 6D). Collectively, these data indicate that Cep57 has a nonredundant role in centriole engagement and cohesion. This conclusion was further strengthened by experiments in which we knocked down Cep57 in *Cep57^+/+^* MEFs (Figure 6, D and E, and Supplemental Figure 5F). The centriole disengagement defect was more profound in *Cep57^T/T^* MEFs than in *Cep57* shRNA-containing MEFs, however, most likely because of their more profound Cep57 insufficiency.

### Cep57 truncation leads to aberrant spindles that missegregate chromosomes.

**Figure 7:**
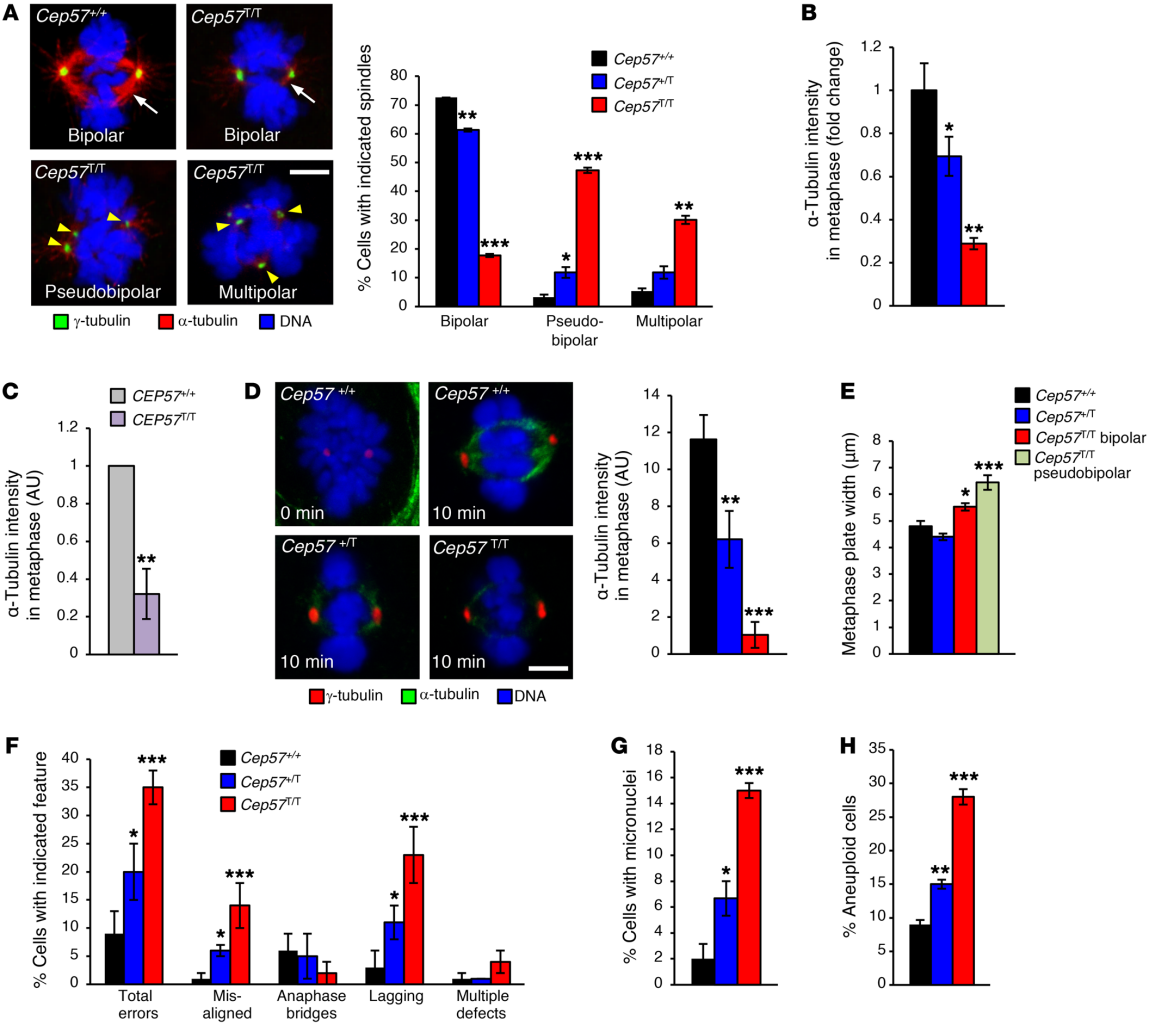
Cep57 truncation leads to aberrant spindles that missegregate chromosomes. (**A**) Representative images of metaphase microtubule configurations and intensity in MEFs. Right: Quantification of spindle abnormalities. (**B**) Quantification of spindle intensities as seen in **A**. (**C**) Quantification of spindle α-tubulin intensity in human fibroblasts of indicated genotypes. (**D**) Microtubule regrowth assay on Cep57-insufficient cells. Left: Images of mitotic MEFs of the indicated genotypes placed on ice for 40 minutes and stained for α- and γ-tubulin after the indicated recovery times at 37°C. Right: Quantification of α-tubulin signals in MEFs of the indicated genotypes. (**E**) Measurement of metaphase plate width in MEFs of indicated genotypes/subgroups. (**F**) Percentage of cells undergoing indicated chromosome missegregation error determined by live-cell imaging on MEFs expressing H2B-mRFP. (**G**) Percentage of cells observed to form micronuclei after a chromosome missegregation event, per analyses performed in **E**. (**H**) Percentage of P5 MEFs with abnormal number of chromosomes counted on metaphase spreads. Polyploid cells were excluded. Analyses in **A**, **B**, and **D** were performed on at least 3 independent lines per genotype (20 cells per line). Analysis in **C** was performed on 1 cell line per genotype (20 cells analyzed per line). The experiment was repeated 3 times. Analyses in **E** and **F** were performed on at least 3 independent lines per genotype (at least 25 cells per line). Analysis in **G** was performed on at least 3 lines per genotype (50 cells per line). Data in **A**–**H** represent the mean ± SEM. Statistical significance in **A**, **E**, and **H** was determined using 1-way ANOVA followed by Tukey’s multiple-comparisons test. Statistical significance in **C**, **D**, **F**, and **G** was determined using a 2-tailed unpaired *t* test, and in **B** using a 1-tailed unpaired *t* test. *P < 0.05, **P < 0.01, ***P < 0.001. Scale bars: 5 μm.

Next, we examined the impact of the observed centrosomal alterations on spindle formation and chromosome segregation. First, we double-labeled *Cep57^T/T^*, *Cep57^+/T^*, and *Cep57^+/+^* MEFs for γ- and α-tubulin to visualize centrosomes and the mitotic spindles. *Cep57^T/T^* metaphases showed high rates of spindle abnormalities, with 47% and 30% of cells having pseudobipolar and multipolar spindles, respectively (Figure 7A). *Cep57^+/T^* MEFs showed the same abnormalities albeit at lower frequencies. Even *Cep57^T/T^* metaphases with bipolar spindles containing normal centrosome numbers were abnormal in that the α-tubulin content was greatly reduced compared with their WT counterparts (Figure 7B). MVA human fibroblasts recapitulated this phenotype (Figure 7C). The α-tubulin content of bipolar spindles in *Cep57^+/T^* MEFs was also significantly reduced, but to a lesser extent than in *Cep57^T/T^* MEFs (Figure 7B). In microtubule regrowth assays, both *Cep57^+/T^* and *Cep57^T/T^* MEFs formed bipolar spindles with significantly reduced α-tubulin contents, indicating that centrosomal microtubule nucleation is impaired in the absence of a full complement of Cep57 (Figure 7D). Several PCM components with known functions in microtubule nucleation and organization, including Cdk5rap2, Pcnt, and Cep152 (49), are present at reduced levels at spindle poles of Cep57-insuffcient cells (Figures 4 and 5), providing a plausible explanation for the above-mentioned spindle defects. Microtubule polymerization rates as determined by EB3-GFP–mediated microtubule plus end growth tracking in live cells were normal in *Cep57*-insufficient MEFs (Supplemental Figure 6A), indicating that microtubule elongation following microtubule nucleation is unperturbed.

Metaphase plates in *Cep57^T/T^* MEFs with normal centrosome numbers were significantly wider than those in *Cep57^+/+^* MEFs (Figure 7E), which is consistent with the dramatic decrease in bipolar spindle microtubule density observed in these cells. The same was true for *Cep57^T/T^* metaphases with pseudobipolar spindles. Spindle length, as measured by the distance between bipolar spindle centrosomes, was unchanged in *Cep57^T/T^* metaphase cells (Supplemental Figure 6B). The timing of centrosome disjunction in late G_2_ phase and the subsequent movement of duplicated centrosomes to opposite poles was also unchanged in *Cep57^T/T^* MEFs with normal centrosome numbers (Supplemental Figure 6C), resulting in the formation of bipolar spindles with normal perpendicular alignment of the spindle to the axis of cell division (Supplemental Figure 6D).

We examined the extent to which the observed spindle abnormalities caused chromosome missegregation by live-cell imaging of H2B-mRFP–expressing *Cep57^T/T^*, *Cep57^+/T^*, and *Cep57^+/+^* MEFs. *Cep57^T/T^* MEFs had markedly increased chromosome missegregation rates compared with *Cep57^+/+^* MEFs, attributed to increases in both misaligned and lagging chromosomes (Figure 7F). A significant proportion of *Cep57^T/T^* MEFs that missegregated chromosomes formed micronuclei (Figure 7G). Similar chromosome segregation defects were observed in *Cep57^+/T^* MEFs, although at lower rates than in *Cep57^T/T^* MEFs. The other 2 MVA-associated proteins, BubR1 and Trip13, are important for sustained mitotic checkpoint activity, the lack of which predisposes cells to erroneous chromosome segregation. However, the mitotic checkpoint was unperturbed in *Cep57^T/T^* MEFs as revealed by nocodazole-challenge assays (Supplemental Figure 7A). Furthermore, recruitment of Mad1 and Mad2 to unattached kinetochores of mitotic chromosomes was normal in both *Cep57^T/T^* MEFs and *CEP57^T/T^* MVA patient cells (Supplemental Figure 7, B and C).

Additionally, the error correction machinery that resolves kinetochore-microtubule malattachments before anaphase onset appeared to function normally in *Cep57^T/T^* MEFs, as assessed by monastrol washout analyses (Supplemental Figure 7D). Progression through mitosis was delayed in *Cep57^T/T^* MEFs even in the absence of segregation errors, indicating that Cep57 is important for proper timing of mitosis (Supplemental Figure 7E). BubR1 also controls mitotic timing, but reduction of BubR1 accelerates rather than inhibits progression (50). Collectively, the above experiments suggest that mitotic spindle abnormalities in *Cep57^T/T^* MEFs are a major cause of microtubule-kinetochore malattachments and chromosome missegregation.

Aberrant chromosome segregation predisposes cells to aneuploidy (51). Indeed, chromosome counts on metaphase spreads revealed a marked increase in the percentage of *Cep57^T/T^* MEFs with abnormal chromosome numbers (Figure 7H and Supplemental Table 1). The same was true for *Cep57^+/T^* MEFs, but rates were much lower. To include cells that may be cell cycle arrested as a result of karyotype abnormalities, we performed complementary experiments in which we used a FISH-based approach to measure copy numbers of chromosomes 4 and 7 in interphase cells. Consistent with the results from chromosome counts, *Cep57^T/T^* MEFs showed significantly increased rates of numerical chromosome abnormalities (Supplemental Figure 7F and Supplemental Table 2). Importantly, *Cep57^T/T^* MEFs with nonmodal FISH signals rarely showed aberrant copy numbers for both chromosomes 4 and 7, confirming data from chromosome counts that tetraploidization is uncommon in these cells.

### Cep57 truncation causes severe MVA in vivo.

**Figure 8:**
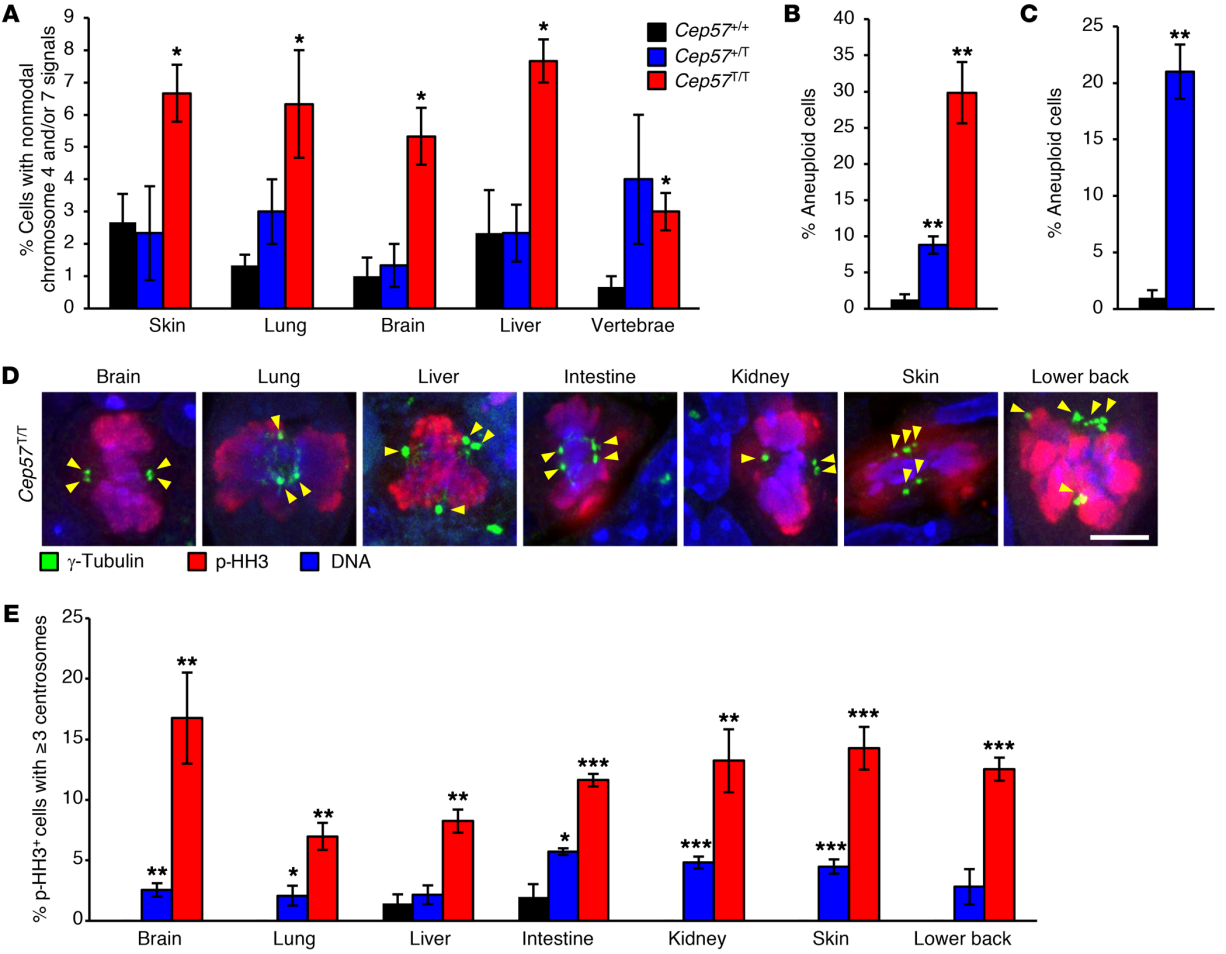
MVA patient–mimetic mice have widespread aneuploidy in vivo. (**A**) Percentage of single cells isolated from specified tissues with aneuploidy as determined by FISH using probes for chromosomes 4 and 7. One-day-old animals were used for the analysis. (**B**) Karyotyping performed on metaphase spreads of cells isolated from the livers of 1-day-old animals. (**C**) Karyotyping performed on metaphase spreads of cells isolated from the spleens of 5-month-old animals. (**D**) Images of p-HH3–positive cells with supernumerary centrosomes of the indicated tissues of 1-day-old *Cep57^T/T^* mice. Scale bar: 5 μm. (**E**) Quantification of mitotic cells with amplified centrosomes in tissues of 1-day-old mice of the indicated genotypes. Analyses in **A**–**C** and **E** were performed on at least 3 animals per genotype. One hundred cells per animal were counted in **A** for each tissue. At least 50 cells per animal were counted in **B** and **C**. Analyses in **E** were performed on at least 50 cells per tissue per animal. Data in **A**–**C** and **E** represent the mean ± SEM. Statistical significance in **A**–**C** was determined using a 2-tailed unpaired *t* test, and in **E** using a 1-tailed unpaired *t* test. *P < 0.05, **P < 0.01, ***P < 0.001.

High aneuploidy rates throughout tissues and organs are a core characteristic of human MVA syndrome. To determine whether this is true in our *Cep57^T/T^* mouse model, we isolated single cells from lung, liver, kidney, skin, brain, and lumbar vertebrae of *Cep57^+/+^* and *Cep57^T/T^* animals and performed FISH for chromosomes 4 and 7 to determine karyotypic instability. All *Cep57^T/T^* organs analyzed had significantly increased numbers of cells with nonmodal chromosome 4 and/or 7 signals, indicative of aneuploid karyotypes (Figure 8A, Supplemental Table 2, and Supplemental Figure 8A). The same analysis on newborn *Cep57^+/T^* pups did not yield evidence for elevated aneuploidy rates. In vivo aneuploidy was further examined by chromosome counts on hematopoietic cells from livers of newborn *Cep57^T/T^* pups, revealing high aneuploidy rates (Figure 8B, Supplemental Table 1, and Supplemental Figure 8B), particularly in comparison with previously reported rates of hematopoietic liver cells of 1-day-old BubR1 hypomorphic mice (8). Importantly, hematopoietic cells from age-matched *Cep57^+/T^* livers also had significantly increased aneuploidy rates, albeit not as high as their *Cep57^T/T^* counterparts (Figure 8B). Furthermore, chromosome counts on metaphase spreads of freshly harvested splenocytes from 5-month-old mice showed that adult *Cep57^+/T^* mice are aneuploidy prone (Figure 8C, Supplemental Table 1, and Supplemental Figure 8C). These observations suggest that the *Cep57^T/T^* and *Cep57^+/T^* models recapitulate the human MVA syndrome to varying degrees.

To determine the extent to which the observed high rates of in vivo aneuploidy in tissues of newborn *Cep57^T/T^* pups correlate with increased centrosome amplification, we quantified centrosome numbers in mitotic cells from a broad spectrum of 1-day-old *Cep57^+/+^* and *Cep57^T/T^* tissues, including brain, lung, liver, intestine, kidney, skin, and lower back soft tissue. Phospho–histone H3 (p-HH3) and γ-tubulin were used to visualize mitotic cells and centrosomes in tissue sections, respectively. Indeed, a substantial proportion of mitotic cells in *Cep57^T/T^* tissues had 3 or more centrosomes, irrespective of the tissue analyzed, whereas such cells were extremely rare or absent in corresponding *Cep57^+/+^* tissues (Figure 8, D and E). Importantly, most tissues from *Cep57^+/T^* mice also showed evidence of centrosome amplification, but at lower rates than observed in *Cep57^T/T^* tissues. Cep57 insufficiency had no discernible impact on the mitotic index of the tissues analyzed (Supplemental Figure 8D). The incidence of apoptosis was also unchanged (Supplemental Figure 8E), suggesting that cell survival was not overtly impacted by the presence of supernumerary centrosomes. Taken together, these results indicate that tissues and organs of *Cep57^T/T^* mice undergo centrosome amplification, albeit at noticeably lower rates than those observed in *Cep57^T/T^* MEFs.

### Cep57 is a haploinsufficient tumor suppressor.

**Figure 9:**
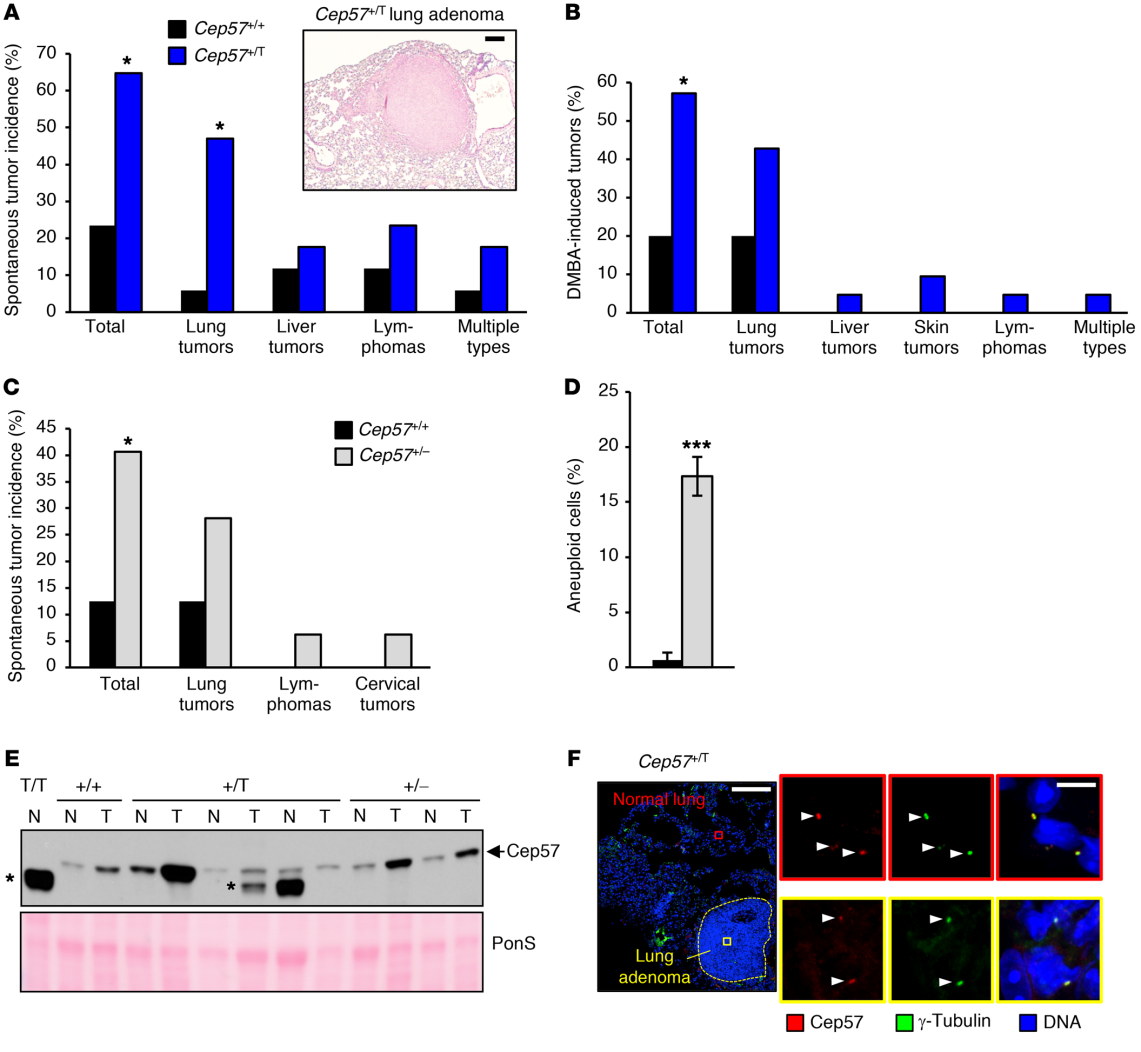
Cep57-insufficient mice are tumor prone. (**A**) Spontaneous tumor incidence in 16-month-old mice (17 *Cep57^+/T^* and 17 *Cep57^+/+^* mice were used). Representative histological image of a lung adenoma from a *Cep57^+/T^* mouse. Scale bar: 1 mm. (**B**) DMBA-induced tumor incidence in 4-month-old mice. Sample sizes of 21 *Cep57^+/T^* and 20 *Cep57^+/+^* mice were used. (**C**) Spontaneous tumor incidence in 16-month-old mice. Sample sizes of 32 *Cep57^+/–^* and 24 *Cep57^+/+^* mice were used. (**D**) Karyotyping performed on metaphase spreads of cells isolated from the spleens of 5-month-old mice. *n* = 3 mice used per genotype. Fifty cells were counted per animal. (**E**) Western blot analysis comparing Cep57 expression between lung adenomas (T) and paired adjacent normal lung tissue (N) lysates from 16-month-old mice of the indicated genotypes to assess *Cep57* loss of heterozygosity. *CEP57^T/T^* (T/T) lung tissue (at P1) was loaded as a control for complete loss of WT Cep57 protein. *Nonspecific band present in some samples. PonS served as loading control. (**F**) Image of a tissue section of a spontaneous lung adenoma with flanking normal tissue from a *CEP57^+/T^* mouse immunolabeled for Cep57 (red) and γ-tubulin (green). Nuclei were visualized with Hoechst. Dotted yellow line marks the tumor. Insets show colocalization of centrosomal Cep57 and γ-tubulin in both normal (red box) and tumor (yellow box) regions. Statistical significance in **A**–**D** was determined using a 2-tailed Fisher’s exact test. *P < 0.05, ***P < 0.001. Scale bar: 100 μm; inset, 5 μm.

MVA syndrome patients with mutations in *BUBR1* and *TRIP13* are at an increased risk for cancer (1, 3). No such predisposition has been reported for the 5 documented patients with biallelic mutations in *CEP57*, the oldest of whom was 15 years of age at the time of publication (52). To determine whether carriers of the *Cep57^T^* mutation are tumor prone, we established cohorts of *Cep57^+/T^* and *Cep57^+/+^* littermates and screened these mice for tumors at 16 months of age. *Cep57^+/+^* mice had a tumor incidence of 24%, which is consistent with tumor rates in control cohorts in previously reported studies (53, 54). In contrast, 65% of *Cep57^+/T^* mice had tumors at 16 months, with lung adenomas being the most prevalent tumor type (Figure 9A). The tumor spectrum was the same between both genotypes, suggesting that *Cep57* mutation accelerates the progression of neoplastic lesions that mice on a 129 × C57BL/6 genetic background normally develop. FISH analysis indicated that *Cep57^+/T^* lung adenomas were significantly more aneuploid than flanking normal lung tissue (Supplemental Figure 9A). Furthermore, centrosome amplification and formation of micronuclei, 2 characteristics of the chromosomal instability phenotype of *Cep57^+/T^* MEFs, occurred at significantly higher rates in *Cep57^+/T^* lung adenomas than in their *Cep57^+/+^* counterparts (Supplemental Figure 9, B and C).

We complemented these spontaneous tumorigenesis studies with a tumor bioassay in which a single dose of the carcinogen 7,12-Dimethylbenz[a]anthracene (DMBA) was applied to the dorsal skin of *Cep57^+/T^* and *Cep57^+/+^* littermate pups at postnatal day 6 (P6). Mice were then analyzed for tumor incidence at 4 months of age. *Cep57^+/T^* mice were highly prone to DMBA-induced tumorigenesis compared with control mice (Figure 9B), although differences in lung tumor size and number did not reach statistical significance (Supplemental Figure 9, D and E).

Our analysis of *Cep57^+/T^* mice did not provide conclusive evidence that reduced *Cep57* expression drives tumorigenesis, because the possibility that the presence of Cep57^T^ protein is a requirement for tumor development cannot be ruled out. We know that truncated Cep57^T^ protein exerts residual biological activity even though it is not detectable by Western blotting or immunolabeling, because, unlike *Cep57^T/T^* mice, *Cep57^–/–^* mice died during embryogenesis and were maternally resorbed before delivery. To address this issue, we generated a cohort of mice heterozygous for a *Cep57*-null allele (*Cep57^+/–^*). At 16 months of age, *Cep57^+/–^* mice showed markedly increased tumor formation compared with *Cep57^+/+^* littermates, with lung adenomas again being the most common tumor type (Figure 9C). The fold increase in tumor incidence was strikingly similar between *Cep57^+/T^* and *Cep57^+/–^* mutant mice. Importantly, *Cep57^+/–^* mice had aneuploidy rates similar to that of *Cep57^+/T^* mice, as evidenced by chromosome counts on splenocytes at 5 months of age (Figure 9D and Supplemental Table 1).

Next, we determined whether the formation of lung tumors in *Cep57^+/T^* and *Cep57^+/–^* mice involved the loss of the remaining WT *Cep57* allele. To this end, we prepared lysates of dissected lung adenomas and corresponding normal lung tissue, and analyzed Cep57 protein levels by Western blot analysis. Lung tumors from both mutants consistently retained Cep57 expression (Figure 9E). In fact, in most tumors, Cep57 expression was elevated compared with normal flanking lung tissue. One possible explanation is that basal Cep57 levels vary among the different cell types of lung tissue, with the cell type undergoing neoplastic transformation expressing relatively high levels of Cep57 compared with nontransformed cell types. Alternatively, cell cycle reentry might result in increased Cep57 expression, with elevated Cep57 levels in tumors perhaps simply reflecting an increase in mitotic index. Importantly, in complementary experiments, we immunolabeled paraffin sections of *Cep57^+/T^* lung tumors and flanking normal tissue for Cep57 and γ-tubulin and determined whether Cep57 was localized at centrosomes. All 7 lung adenomas analyzed showed centrosomal Cep57 labeling in both tumorous and normal lung tissue (Figure 9F and Supplemental Figure 9F). Taken together, these results indicate that Cep57 is a haploinsufficient rather than a classical tumor suppressor.

## Discussion

Mutations in the centrosome-associated protein *CEP57* have been identified in patients with MVA syndrome, but the functions of this protein at the molecular, cellular, and organismal levels remain poorly understood. Using genetically engineered mouse models and primary cells derived from these animals, here we report several important new insights into the biological functions of Cep57.

First, we uncovered a critical biological role for Cep57 in bone development. Several lines of evidence suggest that Cep57 exerts this role through its interaction with Fgf2. Although Fgf2 is implicated in a wide spectrum of biological processes, we find that its expression in newborn pups is largely restricted to osteoblasts, and that the inability of lumbosacral vertebrae to form bone tissue correlates with loss of Fgf2 expression. This, together with earlier observations that Fgf2 is a key regulator of bone formation (39, 40) and that Cep57 and Fgf2 can form a complex in vitro (17, 18), identifies Cep57 as an important modulator of Fgf2 levels in osteoblasts. Even though Cep57 has been shown to serve as a nuclear transporter of Fgf2 in cell culture (17), we did not observe cytoplasmic accumulation of Fgf2 in mouse osteoblasts.

Second, we discovered that Cep57 plays a central role in centrosome maturation, with key components of all toroid PCM layers failing to accumulate as *Cep57^T/T^* cells progress through G_2_ and M. Strikingly, this phenotype was unique to Cep57 and was not observed when either of the Cep57 binding partners Cep63 and Cep152 was depleted. Moreover, *Cep63*-knockout mice are viable and have a normal lifespan, further underscoring the nonredundant roles of Cep57 and Cep63 (23). Importantly, failed centrosome maturation resulted in centrosome amplification, a phenotype that was not linked to changes in Plk4 recruitment to Cep57-deficient centrosomes. On the other hand, centrosome amplification in *Cep57^T/T^* cells was characterized by premature centriole disengagement during metaphase, a phenotype previously reported for Pcnt-cleaved (31, 32, 55) or Cdk5Rap2-depleted cells (30). We find that both these regulators of timely disengagement fail to enrich at Cep57-deficient centrosomes. Since Cdk5rap2 causes supernumerary centrosomes when depleted, a plausible mechanism by which Cep57 acts to prevent centrosome amplification is through the centrosomal recruitment of Cdk5rap2.

Third, we established that *Cep57* is a haploinsufficient tumor suppressor. The observed spontaneous and DMBA-induced cancer phenotypes in *Cep57*-insufficient mice were unexpected because MVA patients with *CEP57* mutations, in contrast to those carrying mutations in *BUBR1* and *TRIP13*, are not known to be predisposed to childhood cancers (1, 3, 6). BUBR1 and TRIP13, but not CEP57, have a common role in the mitotic checkpoint, which has been linked to the separation in cancer phenotypes among MVA syndrome patients (3). Consistent with data from *CEP57* MVA patient fibroblasts (3), MEFs derived from *Cep57^T/T^* mice showed normal mitotic checkpoint activity in a nocodazole-challenge assay. A recent study using epithelial cells instead of fibroblasts reported a role for CEP57 in mitotic checkpoint activity through the recruitment of Mad1-Mad2 to kinetochores of unattached mitotic chromosomes (56). However, we did not observe any defects in kinetochore targeting of Mad1 and Mad2 in *Cep57^T/T^* MEFs or *CEP57^T/T^* patient fibroblasts. One possible explanation that is consistent with all the data is that Cep57 has a cell type–specific role in controlling the mitotic checkpoint.

Given that aneuploidy rates are elevated in *Cep57*-haploinsufficient mice and that cancer is a potential outcome of aneuploidy, it is tempting to speculate that the observed chromosomal instability and cancer phenotypes are causally linked. Tumors developing in *Cep57^+/T^* and *Cep57^+/–^* mice do not undergo loss of heterozygosity, indicating that rates of chromosome segregation during tumorigenesis are unlikely to increase further as a result of a more complete loss of Cep57 functions. Importantly, a key characteristic of the chromosome instability phenotype of *Cep57^+/T^* MEFs, centrosome amplification, is more profoundly present in *Cep57^+/T^* tumors than in *Cep57^+/+^* tumors, supporting the idea that the mechanism of aneuploidization in *Cep57^+/T^* tumors involves microtubule malattachment of chromosomes due to the formation of pseudobipolar spindles. Such spindles produce lagging chromosomes that lead to the formation of micronuclei, which were also observed at increased incidence in *Cep57^+/T^* tumors. Another possible mechanism underlying the increase in attachment errors that cause micronuclei involves centrosomal microtubule nucleation, which we found was also impaired in *Cep57^+/T^* cells. Therefore, even though the chromosomal instability phenotype of *Cep57^+/T^* mice is considerably milder than that of *Cep57^T/T^* mice, the degree of aneuploidization might be optimal for driving neoplastic transformation, thereby obviating the need for loss of heterozygosity. Mutant mice that are subject to centrosome amplification due to *Plk4* overexpression are also tumor prone (38), providing broader support for the notion that supernumerary centrosomes are causally implicated in neoplastic transformation. Additional mechanisms by which Cep57 insufficiency may drive tumorigenesis cannot be excluded. For instance, in cell culture CEP57 has been shown to maintain a quiescent state by restricting nuclear import of cyclin D1, raising the possibility that CEP57 insufficiency promotes uncontrolled cell proliferation by precociously driving cells into S phase (57). Furthermore, a role for Cep57 in the control of cell signaling pathways beyond the Fgf2 pathway cannot be excluded at this point and needs to be further explored in future studies.

Finally, a remarkable difference between *CEP57^T/T^* patients and *Cep57^T/T^* mice is the impact of the biallelic mutation on postnatal viability, particularly given that the mitotic phenotypes of fibroblasts derived from a *CEP57^T/T^* patient and the mimetic mouse cells share striking similarities. It is difficult to pinpoint the exact cause of death of *Cep57^T/T^* mice shortly after birth, because defects in both bone development and karyotypic stability are severe. Our conclusion that the chromosomal instability phenotype is severe is based on direct comparisons of aneuploidy rates in hematopoietic cells in livers from 1-day-old *Cep57^T/T^* and *BubR1^–/H^* mice, both of which die at birth with 30% and 21% aneuploidy (8), respectively. *BubR1^–/H^* mice do not exhibit gross defects in skeletal development (8), suggesting that the severe chromosomal instability (CIN) phenotype may be sufficient to compromise viability of the 1-day-old *Cep57^T/T^* mice irrespective of defects in bone development. An attempt to bypass premature postnatal death by inactivating p53 in *Cep57^T/T^* mice failed, with double mutants showing no phenotypic improvement (data not shown).

In summary, we discovered that Cep57 is implicated in 2 diverse biological processes, bone formation and tumor suppression. Cep57 profoundly impacts the levels of Fgf2, a key regulator of ossification, in osteoblasts during embryonic development. Cep57 is also a master regulator of centrosome maturation, a function that we find is imperative for the assembly of mitotic spindles with proper microtubule-kinetochore attachments that allow for accurate segregation of duplicated chromosomes. Cep57 insufficiency could drive tumorigenesis in this way. Interestingly, *CEP57* insufficiency correlates with tumor growth and metastasis in 2 prostate cancer patient cohorts, and monoallelic mutation was reported in early-onset familial prostate cancers (58, 59). Irrespective of the exact mechanism of tumorigenesis, the currently available data warrant future investigations as to whether homozygous and heterozygous carriers of inactivating *CEP57* mutations would benefit from intensified cancer screenings, for instance by the use of newly developed multianalyte blood tests (60).

## Methods

### Mouse strains.

All mice used in this study were housed in a pathogen-free barrier facility. *Cep57^+/T^* mice were generated using TALEN-mediated gene targeting. Homology arms spanning 809 bp in intron 8 and 985 bp in intron 9 were used for targeted insertion of donor template consisting of 11 bp (CAATGTTCAGC) duplication in exon 9 of *Cep57* along with a neomycin selection cassette flanked by loxP sites placed 5′ of exon 9. Embryonic stem cells were injected, yielding 3 founders, 2 of which were used for experiments after verification of germline transmission. The experiments were performed on a 129/Sv × C57BL/6 background. To generate *Cep57^+/–^* mice, we deleted exons 1–4 using CRISPR/Cas9 technology in fertilized eggs. Two founder lines with confirmed deletion of Cep57 exons 1–4 were maintained on an FVB/N background and used for experiments.

### Tumor analysis.

For spontaneous tumor analysis, mutant and control mice were housed together and aged for 16 months. Mice were monitored daily for wellbeing. At the end of the study, mice were sacrificed and grossly examined for overt tumors. Tumor characteristics were ascertained by histological analysis. For carcinogen-induced tumorigenesis, 50 μl of 0.5% DMBA in acetone was applied to the dorsal skin on P6, and mice were sacrificed 4 months later. All tumors were imaged, and sizes were estimated by measurement of the longest diameter.

### Generation of Cep57 N-terminus antibody.

A region corresponding to Cep57 amino acids 1–252 was subcloned into a GST expression vector (pGEX4T-3). The GST-tagged Cep57 fragment was expressed in *E*. *coli* RIL cells, purified using glutathione Sepharose beads, and concentrated using a spin column. The resulting protein was used as an antigenic peptide for inoculation of 2 rabbits for polyclonal antibody production (Cocalico Biologicals). Exsanguination bleed serum was affinity-purified using GST-Cep57 (1–252) recombinant protein. The Cep57 plasmid pUBhrGFP_mCep57 was a gift from Ko Momotani (University of Virginia, Charlottesville, Virginia, USA).

### Generation of centrin-tdTomato.

Mouse centrin 2 cDNA (mr201474) was purchased from Origene and ligated into the Xho1 and Xma1 sites of the pLVX-tdTomato vector from Clontech (catalog 632564).

### Histological analysis.

One-day-old mice were fixed for 24 hours in Bouin’s fixative and processed for histological analysis by serial sagittal sectioning. To visualize bone and cartilage, skin and internal organs excluding the brain were carefully removed from 1-day-old mice and fixed overnight in 95% ethanol. Cartilage was stained for 24 hours using 0.2 mg/ml Alcian blue (Sigma-Aldrich A5268) in 4:1 solution of 95% ethanol/acetic acid. Pups were washed twice with 95% ethanol, and soft tissues were digested in 2% KOH for 7 hours. Next, bones were stained using 1% KOH, 75 μg/ml alizarin red (Ricca Chemical, catalog 500-16), overnight and then destained using 1% KOH/20% glycerol for 2–3 weeks with solution changes daily. The stained embryos were imaged using a light microscope (Olympus SZX12).

### Cell culture.

*Cep57^+/+^*, *Cep57^+/T^*, *Cep57^T/T^*, and *Cep57^+/–^* MEFs were generated and cultured as previously described (61). Primary MEFs were frozen at P2 or P3 and used for experimentation between P4 and P6. At least 3 independently generated MEF lines per genotype were used. Primary cultures were maintained in fully supplemented DMEM containing 10% FBS. Patient skin fibroblasts homozygous for *CEP57* 915_925dup11 (MVA T43102) were generated by Rahman’s laboratory (The Institute of Cancer Research). *CEP57^+/+^* human skin fibroblasts were obtained from age-matched healthy individuals.

### Karyotype analysis and interphase FISH.

Chromosome counts of metaphase spreads were performed as previously described (61). Interphase FISH of chromosomes 4 and 7 was performed as described previously (62).

### Live-cell imaging.

Chromosome segregation analysis, mitotic progression analysis, cytokinesis completion, and nocodazole-challenge assays were performed on MEFs stably expressing H2B-mRFP, as previously described (63).

### Western blot analysis.

Western blot analyses were performed as described previously (63). Primary antibodies used for Western blotting were mouse anti–β-actin (clone AC-15, 061M4808, 1:40,000; Sigma-Aldrich), rabbit anti-Fgf2 (TA321421, 1:2,000; Origene), rabbit anti–Cep57 (1–252) (in house, 1:500), and rabbit anti–Cep57 C-terminus (1:10,000; gift from Ko Momotani, University of Virginia, Charlottesville, Virginia, USA). See complete unedited blots in the supplemental material.

### Immunohistochemistry.

Freshly harvested tissues were fixed in 4% paraformaldehyde (PFA) overnight, and IHC was performed using a SignalStain Citrate unmasking solution (Cell Signaling, 14746) and SignalStain Boost detection reagent (Cell Signaling, 8114) according to the manufacturer’s instructions. Primary antibodies used were rabbit anti-Fgf2 (SC 365106, 1:200; Santa Cruz Biotechnology), rabbit anti-pAkt^308^ (CST2965, 1:250; Cell Signaling), and rabbit anti–p-Erk (CST9101, 1:100; Cell Signaling).

### Indirect immunofluorescence and confocal microscopy.

Immunofluorescence was performed as previously described (63). For centrosome staining, cells were fixed in PHEM (25 mM HEPES, 10 mM EGTA, 60 mM PIPES, and 2 mM MgCl_2_ at pH 6.9) for 5 minutes at room temperature (RT) followed by ice-cold methanol for 10 minutes. For microtubule staining, cells were fixed in 4% PFA for 5 minutes at RT. For kinetochore staining, cells were fixed in 1% PFA for 5 minutes. Cells were then permeabilized in 1× PBS/0.2% Triton X-100 for 15 minutes, and blocked in 1× PBS/5% BSA for 60 minutes at RT. Spindle geometry and centrosome separation (63) studies were performed as previously described. For staining tissue sections, freshly harvested tissues were fixed in 4% PFA overnight, and immunofluorescence was performed as previously described (64). Primary antibodies used were rabbit anti–γ-tubulin (T5192, 1:300; Sigma-Aldrich), rabbit anti–p-HH3 (Ser10) (06-570, 1:100; EMD Millipore), and rabbit anti-Fgf2 (TA321421, 1:300; Origene). A laser-scanning microscope (LSM 880; Carl Zeiss) with an inverted microscope (Axiovert 100M; Zeiss) was used to analyze immunostained cells and capture images. Quantification of signals was carried out as previously described (63). Primary antibodies used for immunostaining were: mouse anti–γ-tubulin (T6557/clone GTU-88, 1:300; Sigma-Aldrich), rabbit anti–γ-tubulin (T5192, 1:300; Sigma-Aldrich), mouse anti–α-tubulin (T9026/clone DM1A, 1:1,000; Sigma-Aldrich), rabbit anti–p-HH3 (Ser10) (06-570, 1:1,000; EMD Millipore), rabbit anti-Plk4 (1:1,000; provided by Andrew Holland, Johns Hopkins University, Baltimore, Maryland, USA), rabbit anti-Cep192 (1:500; provided by Laurence Pelletier, University of Toronto, Toronto, Ontario, Canada), rabbit anti-Pcm1 (NB100-58829, 1:500; Novus Biologicals), mouse anti–centrin 2 (1:4,000; provided by Jeffrey Salisbury, Mayo Clinic, Rochester, Minnesota, USA), rabbit anti–Cep57 N-terminus (1:500, in house), rabbit anti-Cep63 (06-1292, 1:500; EMD Millipore), rabbit anti-Cep152 (GTX 128027, 1:500; GeneTex), rabbit anti-pericentrin (1:500; Abcam ab4448), rabbit anti–c-Nap1 (1:500; provided by Kunsoo Rhee, Seoul National University, Seoul, Korea), rabbit anti-Cdk5rap2 (06-1398, 1:1,000; EMD Millipore), rabbit anti-Mad1 (1:1,000, in house; ref. 65), rabbit anti-Mad2 (1:1,000, in house; ref. 65), rabbit anti-cenexin (1:200; Abcam ab43840), rabbit anti-centrobin (1:500; Abcam ab70448), human anti-centromeric antibody (1:100, 15-234-0001; Antibodies Inc.). To detect mouse anti–γ-tubulin in mouse tissues, an IgG-specific secondary antibody was used.

### Centriole disengagement assays.

Two methods were used to assess centriole disengagement. The main method was slightly adapted from a previously described protocol (66). Briefly, cells were labeled for centrin 2 and Hoechst to visualize centrioles and chromosomes, respectively. Cells with a single pair of centrioles on each side of the metaphase plate were identified, and the distance between paired centrioles was measured and plotted. A distance between paired centrioles greater than 1 μm was used as a measure for centriole disengagement. The second method was performed as previously described and is based on c-Nap1 and centrin 2 immunolabeling (47).

### Quantitative reverse transcriptase PCR.

Quantitative reverse transcriptase PCR was performed as previously described (63). Primer sequences used were as follows: Cep57: forward 5′-AGCACAATCAAGAACTGGCATC-3′, reverse 5′-TTCCAGCTGGCTCTGAACAT-3′; Fgf2 q1: forward 5′-TTCATAGCAAGGTACCGGTTG-3′, reverse 5′-AGAAGAGCGACCCACACG-3′; Fgf1: forward 5′-GGTTGTGATCTCCCCTTCAG-3′, reverse 5′-AGTGGAGTGAAGAGAGCCCC-3′; housekeeping gene GAPDH: forward 5′-TGCACCACCAACTGCTTA-3′, reverse 5′-TGGATGCAGGGATGATGTTC-3′; Cep57 C-terminus: forward 5′-GTTCGAAAATACCAAGCCCAG-3′, reverse 5′-TTCTTACTTGCGGTCCTTGG-3′; Cep63: forward 5′-CTCCTCCCTATGGGACTCTGG-3′, reverse 5′-CGAACTTCGGCTTTTCCATCTT-3′; Cep152: forward 5′-CAGCAGCTACTCACAGACCTC-3′, reverse 5′-TATCCCGCTCAATCCACTTCC-3′; Pcnt: forward 5′-AGACATACCAAGAAGACCTGACT-3′, reverse 5′-ACACAGGACGAACTGATTTCTG-3′; Cdk5rap2: forward 5′-TCCCTGGCTAGGAAAGCTGAG-3′, reverse 5′-TCCTGGTTGTGTCTCTCCTTC-3′.

### Cell cycle and cell death analysis.

Propidium iodide staining was used for cell cycle analysis according to the manufacturer’s protocol (BD Biosciences). Cell death in tissue sections was quantified after staining with TUNEL/Hoechst (Roche).

### Microtubule growth tracking.

EB3-pEGFP (provided by Mark McNiven, Mayo Clinic, Rochester, Minnesota, USA) was subcloned into pTSIN lentiviral vector. Cells stably expressing EB3-GFP were seeded onto glass-bottom dishes (MatTek). Images were taken every 2 seconds using an LSM780 Zeiss microscope system with CO_2_ Module S, TempModule S, Heating Unit XL S, Camera (AxioCam MRm; Zeiss) and Zen software (Zeiss). Imaging medium was kept at 37°C.

### Microtubule regrowth assay.

Cells were grown in the presence of 25 μM monastrol for 4 hours, washed 3 times with culture medium, and placed on ice-cold media supplemented with 10 mmol/l HEPES (pH 7.25) for 40 minutes to depolymerize microtubules. Cells were then transferred to prewarmed medium at 37°C for 10 minutes to allow microtubule regrowth and fixed immediately with ice-cold MeOH for 10 minutes and immunostained for γ- and α-tubulin.

### Lentiviral transduction.

The nontargeting shRNA TRC2 negative control (SCH202) and Cep57 shRNA (TRC1 and TRC1.5) clones NM-026665.2-1210s1c1, NM-026665.2-1284s1c1, NM-026665.2-1383s1c1, NM-026665.2-2291s1c1, and NM-026665.2-270s1c1; Cep63 shRNA (TRC2) clones NM-001081122.1-1581s21c1, NM-001081122.1-1997s21c1, NM-001081122.1-2434s21c1, NM-001081122.1-400s21c1, and NM-001081122.1-848s21c1; Cep152 shRNA (TRC2) clones NM-001081091.1-3281s21c1, NM-001081091.1-4188s21c1, NM-001081091.1-4275s21c1, NM-001081091.1-5514s21c1, and NM-001081091.1-765s21c1; Cdk5rap2 shRNA (TRC2) clones NM-145990.3-2273s21c1, NM-145990.3-3829s21c1, NM-145990.3-388s21c1, and NM-145990.3-4292s21c1; and pericentrin shRNA (TRC2) clones NM-008787.3-1482s21c1, NM-008787.3-6654s21c1, NM-008787.3-742s21c1, NM-008787.3-8637s21c1, and NM-008787.3-9047s21c1 were purchased from Sigma-Aldrich; and lentiviruses were prepared according to the manufacturer’s instructions. Cells were infected with targeted shRNA or nontargeting shRNA for 48 hours, selected with puromycin (2 μg/ml) for 48 hours, and used for experiments the following day. The full set of shRNA was checked for efficient knockdown. The following shRNAs showed strong knockdown: Cep63 shRNAs 1 and 2 (in combination); Cep152 shRNA 1; PCNT shRNAs 2 and 4 (in combination); Cdk5rap2 shRNAs 1; Cdk5rap2 shRNAs 3, and 4 (in combination); Cep57 shRNA 2; and Cep57 shRNA 3.

### Statistics.

GraphPad Prism software was used for all statistical analyses. Statistical significance for comparisons was determined by either 2-tailed, unpaired *t* test; 1-way ANOVA followed by Tukey’s multiple-comparisons test; 2-tailed Fisher’s exact test; or 1-tailed Fisher’s exact test with Bonferroni’s correction. A *P* value less than 0.05 was considered statistically significant. Sample sizes for spontaneous tumor studies and DMBA-induced tumor studies were chosen on the basis of power analysis and previously published studies in which differences were observed. No samples were excluded. The experiments were not randomized, and the investigators were not blinded.

### Study approval.

All animal procedures were reviewed and approved by the Institutional Animal Care and Use Committee of the Mayo Clinic, Rochester, Minnesota (protocol A00001648).

## Author contributions

KA helped design and conducted most of the experiments and acquired and interpreted data. CJS, KBJ, ROFV, BAD, and DJK performed experiments and interpreted data. MH generated the Cep57 mutant mouse strains. NR established the *CEP57* MVA patient cell line used. KA, DJK, and JMVD wrote the manuscript, and all authors edited the manuscript. JMVD directed and supervised all aspects of the study.

## Supplementary Material

Supplemental data

## Version 1 07/23/2018

Electronic publication

## Version 2 08/01/2018

Print issue publication
