# Supplementary material for: Mosaic-variegated aneuploidy syndrome mutation or haploinsufficiency in Cep57 impairs tumor suppression
Source: J Clin Invest. 2018 Jul 23;128(8):3517–34. doi: 10.1172/JCI120316 (PMC6063474; doi:10.1172/JCI120316)
Supplement: Supplemental data [file jci-128-120316-s269.pdf]

A

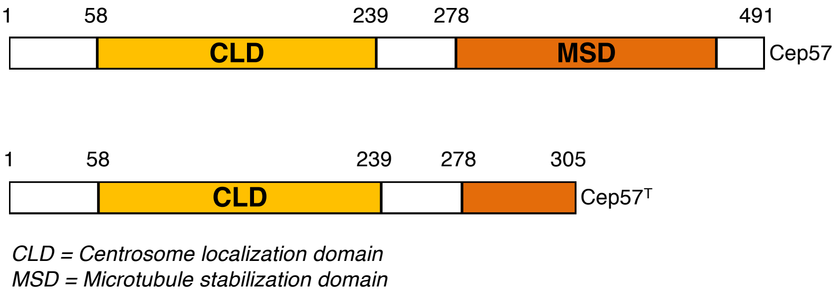

B

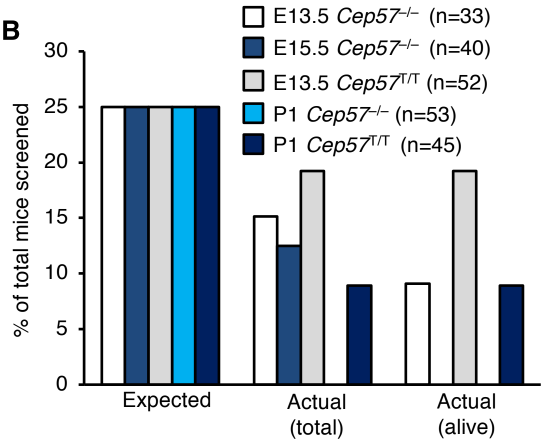

C

	Cep57+/+ (n=5)	*Cep57*T/T (n=5)
Bifid vertebral body ossification	0/5	5/5
Splayed pedicles	0/5	5/5
Vertebral hypoplasia	0/5	5/5
Last vertebral body ossified	Ct14	S4

D

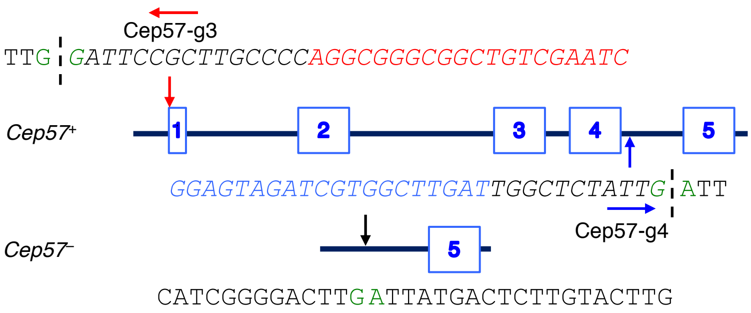

E

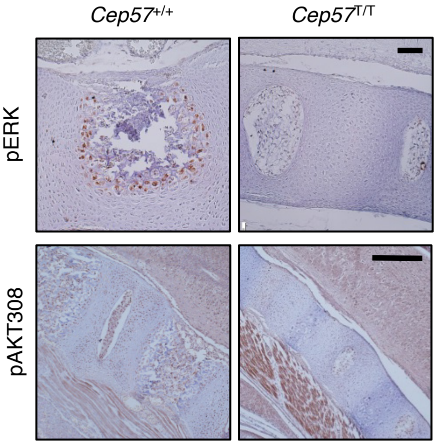

F

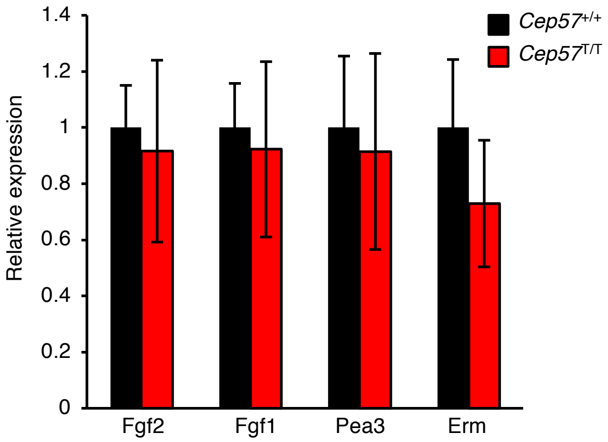

Supplemental Figure 1. Cep57 controls bone development and is essential for embryogenesis. (A) Domain structures of the wildtype and the truncated Cep57 proteins. **(B)** Percentage of mice acquired with indicated genotype at E13.5, E15.5 or postnatal-day-1 and survival status. **(C)** Summary of observed vertebral defects in wildtype and *Cep57*$^{T/T}$ mice stained with Alizarin red and Alician blue to visualize bone and cartilage, respectively. Vertebral hypoplasia was qualitatively assessed. Terminal vertebral body ossified was tabulated separately for each mouse analyzed and the average is represented. **(D)** Gene targeting strategy for creating *Cep57*$^{-}$ allele. Shown are the wildtype allele with guide RNAs designed to target exons 1-4 (top) and the (–) allele after recombination (bottom). **(E)** Immunohistochemical staining for pErk and pAkt performed on sagittal sections of 1-day-old mice of indicated genotypes. At least 3 animals/genotype were used. **(F)** qPCR analysis of indicated transcripts in vertebral bone cDNA of indicated genotypes. *Gapdh* was used as housekeeping gene. Data in **F** represent the mean ± SEM. At least 3 animals/genotype were used. Statistical significance in **F** was evaluated using a 2-tailed unpaired *t* test. Scale bars: 10 μm.

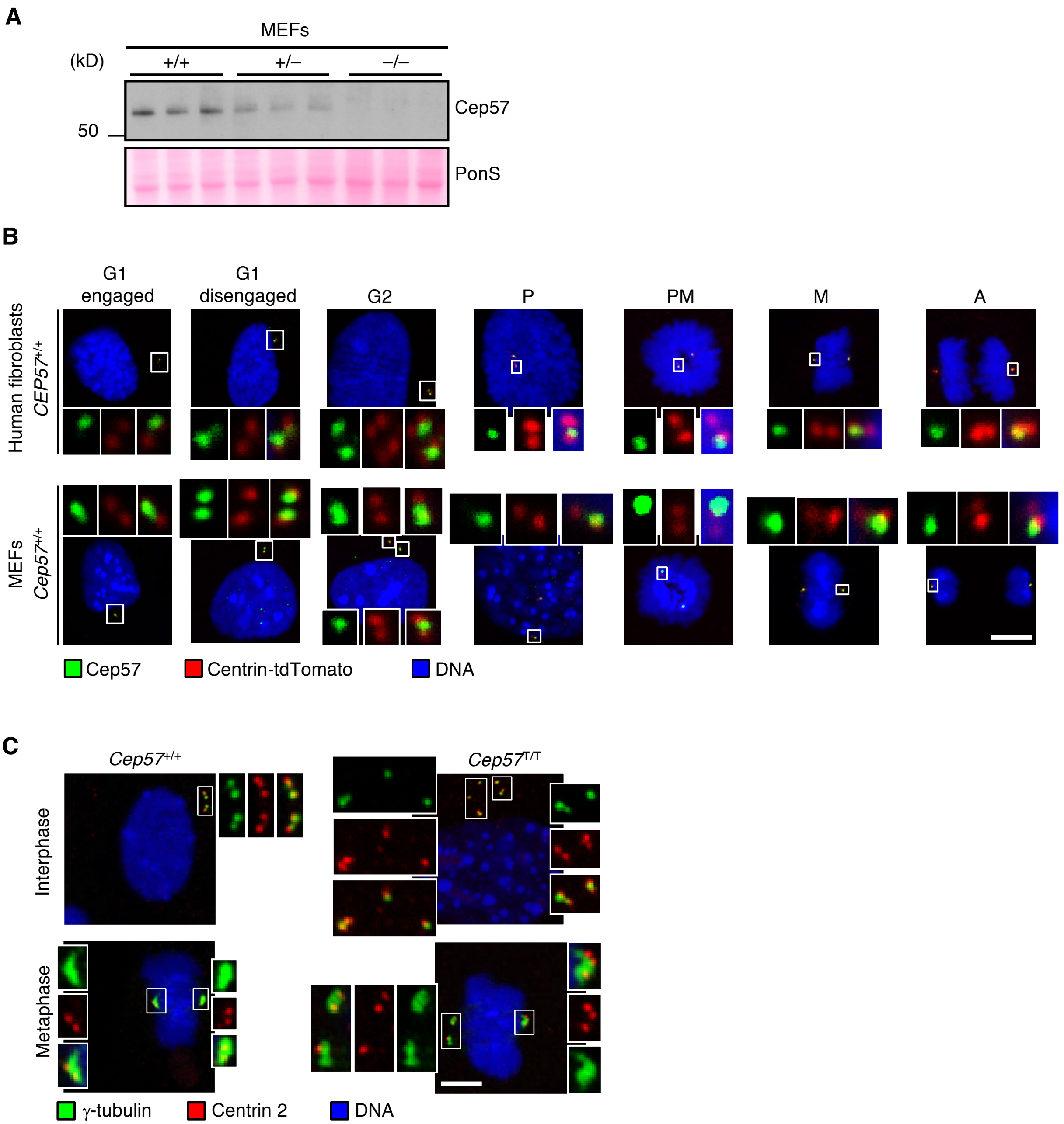

A
MEFs
(kD)
+/+
+/–
–/–
Cep57
50
PonS
B
G1 engaged
G1 disengaged
G2
P
PM
M
A
Human fibroblasts CEP57+/+
MEFs Cep57+/+
Cep57
Centrin-tdTomato
DNA
C
Cep57+/+
Cep57T/T
Interphase
Metaphase
γ-tubulin
Centrin 2
DNA

Supplemental Figure 2. Cep57 localizes to centrosomes throughout the cell cycle and its truncation promotes centrosome amplification. (A) Western blot of MEF lysates comparing Cep57 expression levels among the indicated genotypes. PonS served as loading control. **(B)** Representative images of wildtype human fibroblasts and MEFs in G_1, G_2, prophase (P), prometaphase (PM), metaphase (M) and anaphase (A) phases of the cell cycle. Cells stably expressed centrin 2-tdTomato to visualize centrioles and were immunolabeled for Cep57 (green) to assess its centrosomal localization. We note that Cep57 localizes in a single centrosomal spot early in G_1, while localization to both centrioles is observed after centriolar disengagement in mid/late G_1. **(C)** Representative images of MEFs with indicated genotypes immunolabeled for γ-tubulin and centrin 2.

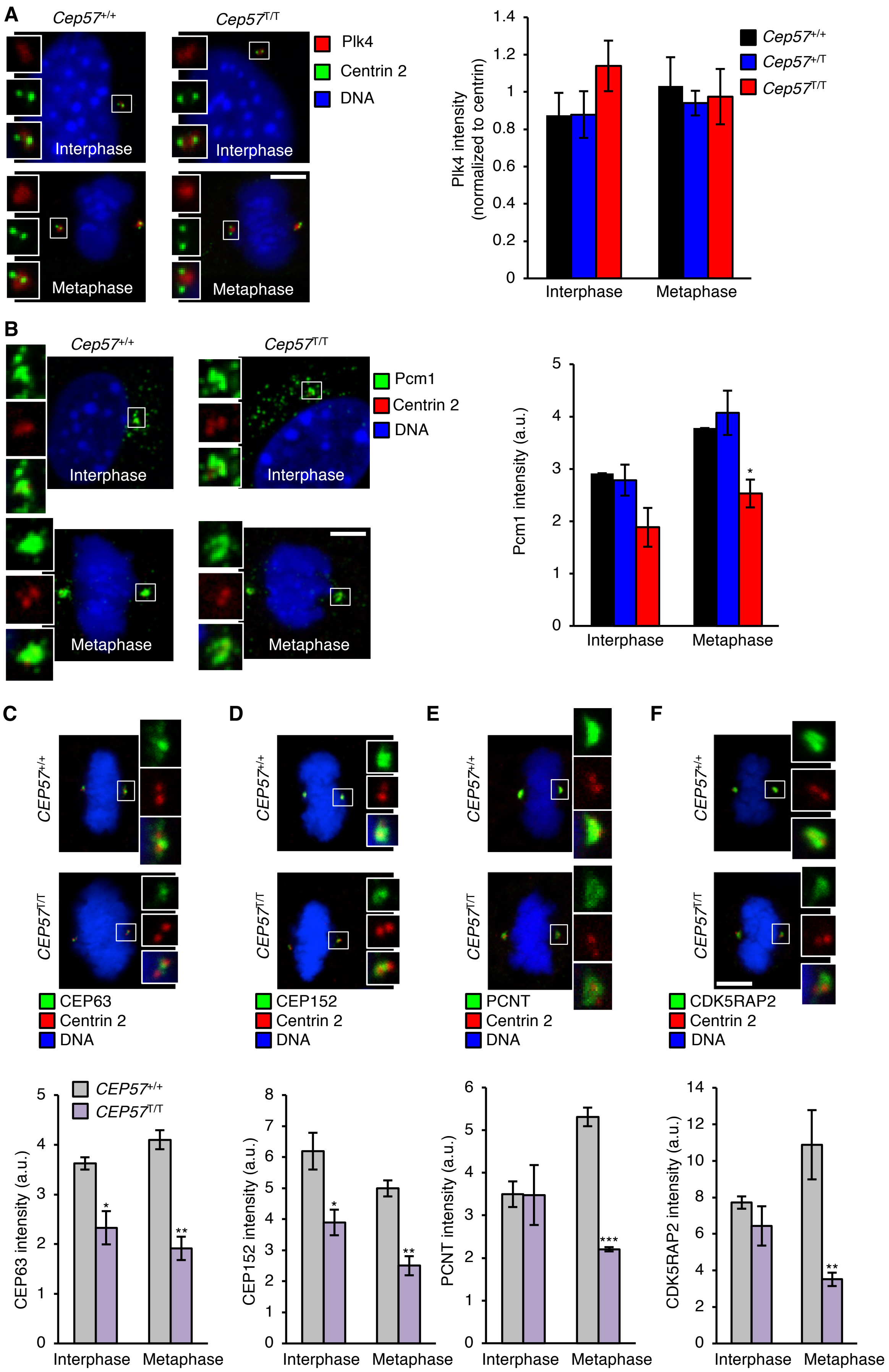
A
Cep57+/+
Cep57T/T
Plk4
Centrin 2
DNA
Interphase
Metaphase
Plk4 intensity (normalized to centrin)
1.4
1.2
1
0.8
0.6
0.4
0.2
0
Cep57+/+
Cep57+/T
Cep57T/T
B
Pcm1
Centrin 2
DNA
Pcm1 intensity (a.u.)
5
4
3
2
1
0
*
C
D
E
F
CEP57+/+
CEP57T/T
CEP63
CEP152
PCNT
CDK5RAP2
CEP63 intensity (a.u.)
CEP152 intensity (a.u.)
PCNT intensity (a.u.)
CDK5RAP2 intensity (a.u.)
*
**
***

Supplemental Figure 3. Cep57 truncation perturbs centrosome maturation. (A) Representative images from interphase and metaphase MEFs immunolabeled for Plk4 and centrin 2. Quantification of Plk4 signal intensity normalized to centrin 2 is shown on the right. **(B)** Representative images from interphase and metaphase MEFs labeled for Pcm1 and centrin 2. Quantification of Pcm1 signal intensity is shown on the right. **(C-F)** Representative images of metaphase human fibroblasts stained for the corresponding protein and centrin 2 (top). Quantification of signal intensities of CEP63, CEP152, CDK5RAP2 and PCNT in human fibroblasts (wildtype control and MVA patient fibroblasts) are shown (bottom). Analyses in **A**-**B** were performed on at least 3 independent lines/genotype (20 cells/line). Analyses in **C**-**F** were performed on 1 cell line/genotype, 20 cells/line. The experiment was repeated 3 times. Data represents mean ± SEM. Statistical significance in **A** was determined using 1-way ANOVA, followed by Tukey's multiple comparisons test. Statistical significance in **B**-**F** was determined using a 2-tailed unpaired *t* test. $^*P < 0.05$, $^{**}P < 0.01$, $^{***}P < 0.001$. Scale bars: 5 μm.

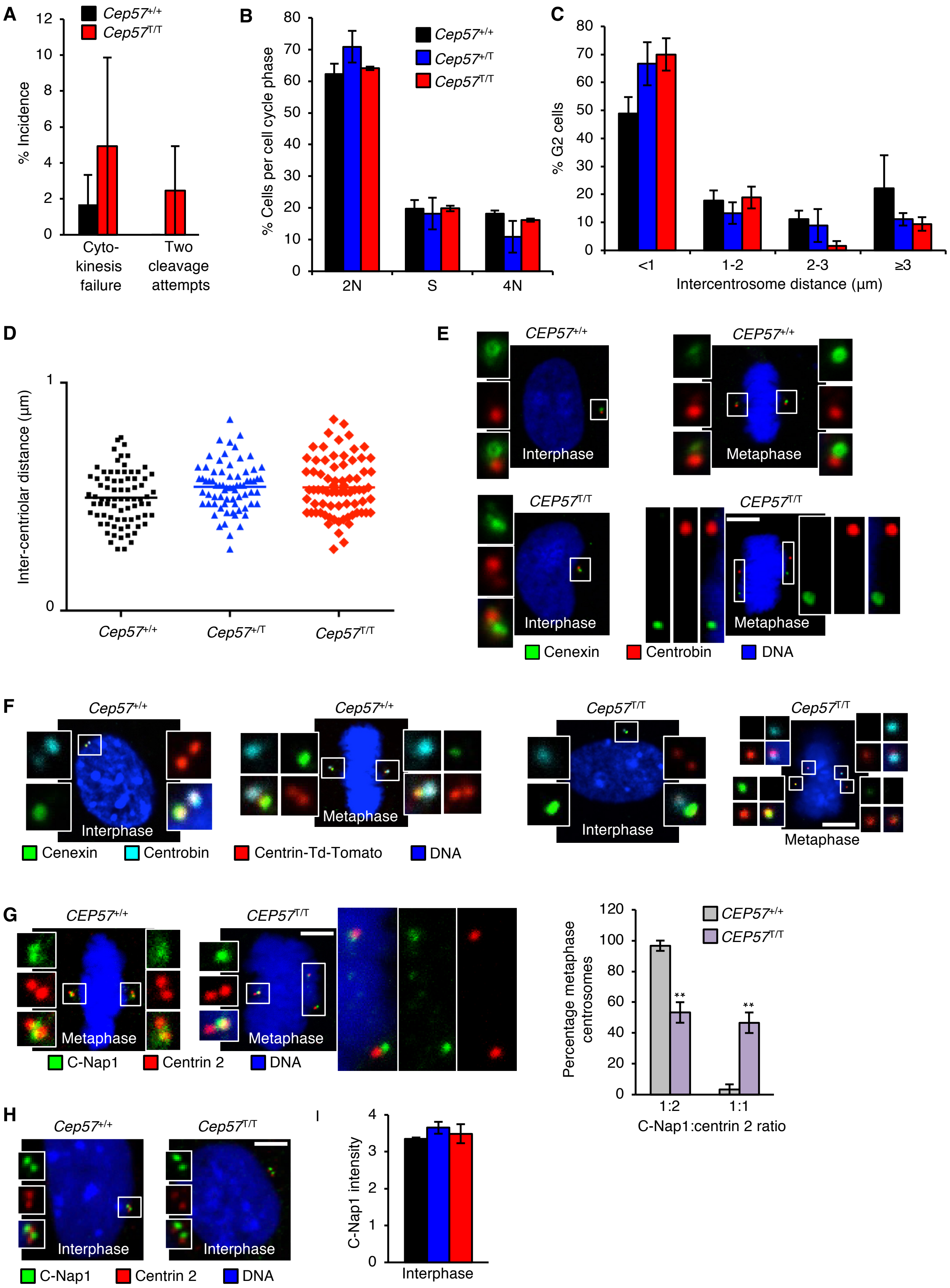
A
B
C
D
E
F
G
H
I
% Incidence
Cyto-kinesis failure
Two cleavage attempts
% Cells per cell cycle phase
2N
S
4N
% G2 cells
Intercentrosome distance (µm)
<1
1-2
2-3
≥3
Inter-centriolar distance (µm)
Cep57+/+
Cep57+/T
Cep57T/T
CEP57+/+
CEP57T/T
Interphase
Metaphase
Cenexin
Centrobin
DNA
Centrin-Td-Tomato
C-Nap1
Centrin 2
Percentage metaphase centrosomes
C-Nap1:centrin 2 ratio
1:2
1:1
C-Nap1 intensity

Supplemental Figure 4. **Cep57 insufficiency causes premature centriole disengagement.** **(A)** Cytokinesis completion analyzed by live cell imaging on H2B-mRFP overexpressing MEFs followed from nuclear envelope breakdown until cytokinesis completion. Cytokinesis was observed by DIC imaging. **(B)** Cell cycle analysis performed on passage 5 MEFs by propidium iodide staining. **(C)** Centrosome splitting analysis performed on G_2 cells identified by immunofluorescence labeling for pHH3 and centrin 2. Inter-centrosomal distance measurements are shown for corresponding genotypes. **(D)** G_2 inter-centriolar distance in MEFs of the indicated genotypes. **(E)** Representative images of wildtype and MVA human fibroblasts at the indicated cell cycle stages stained for cenexin and centrobin. **(F)** Representative images of interphase and metaphase MEFs of indicated genotypes stably expressing centrin 2-tdTomato and stained for cenexin and centrobin. **(G)** (Left) Representative images of wildtype and MVA human fibroblasts in metaphase stained for C-Nap1 and centrin 2. (Right) Quantification of cells with the indicated C-Nap1 and centrin 2 ratio. **(H)** Representative images showing c-Nap1:centrin 2 ratio in interphase MEFs of indicated genotypes. **(I)** C-Nap1 intensity measured in interphase cells with normal centrosome number. Analyses in **A**-**F** were performed on at least 3 independent lines/genotype (20-25 cells/line). Data in **A-C, G** represent the mean ± SEM. Analysis in **G** was repeated 3 times. Statistical significance in **A** and **G** were determined using a 2-tailed unpaired *t* test. Statistical significance in **B**-**D** and **I** were determined using a 1-way ANOVA, followed by Tukey's multiple comparisons test. $^{**}P < 0.01$. Scale bars: 5 μm.

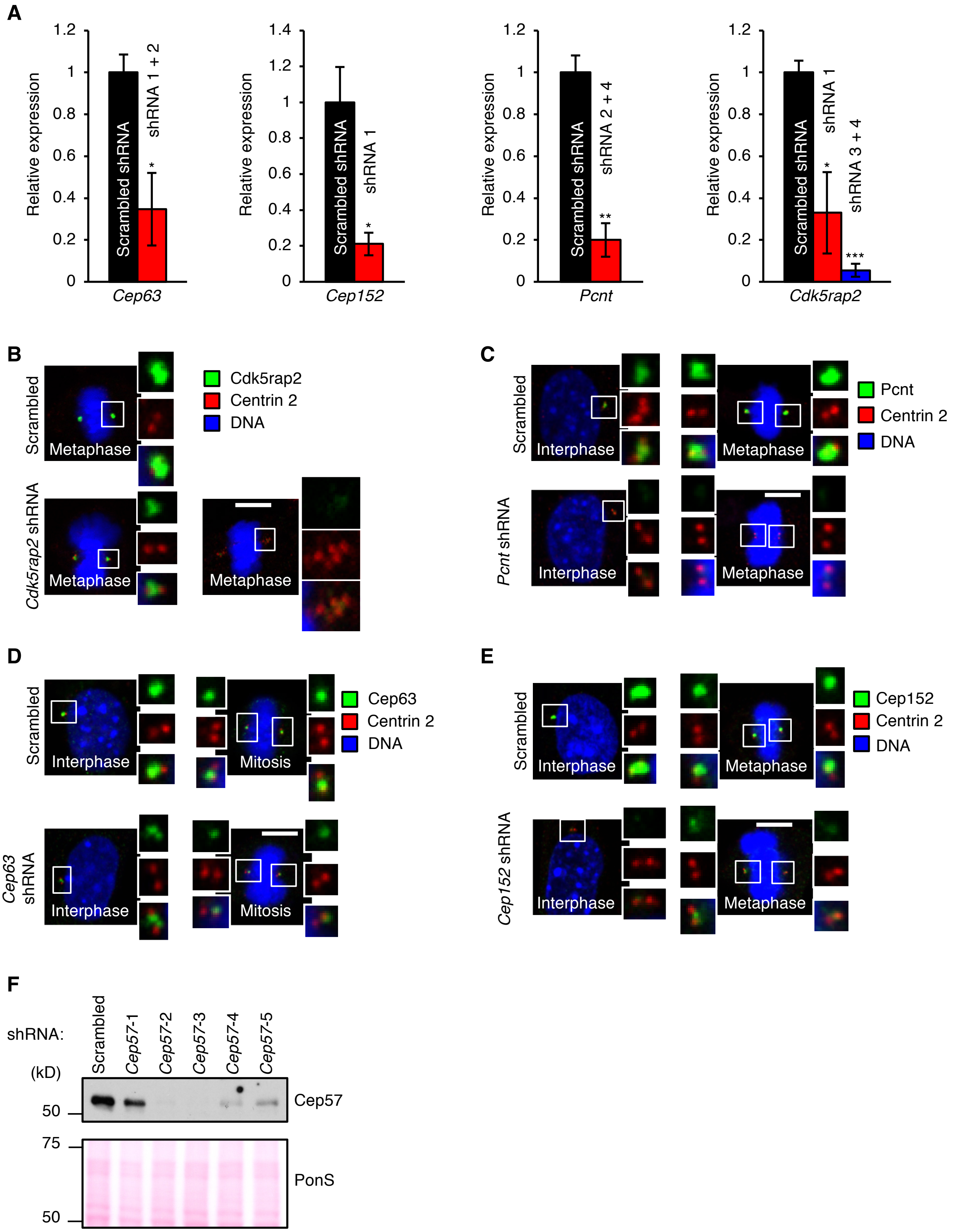
A
Relative expression
Scrambled shRNA
shRNA 1 + 2
Cep63
shRNA 1
Cep152
shRNA 2 + 4
Pcnt
shRNA 1
shRNA 3 + 4
Cdk5rap2
B
Scrambled
Cdk5rap2 shRNA
Metaphase
Cdk5rap2
Centrin 2
DNA
C
Scrambled
Pcnt shRNA
Interphase
Metaphase
Pcnt
Centrin 2
DNA
D
Scrambled
Cep63 shRNA
Interphase
Mitosis
Cep63
Centrin 2
DNA
E
Scrambled
Cep152 shRNA
Interphase
Metaphase
Cep152
Centrin 2
DNA
F
shRNA:
Scrambled
Cep57-1
Cep57-2
Cep57-3
Cep57-4
Cep57-5
(kD)
50
75
50
Cep57
PonS

Supplemental Figure 5. **Premature centriole disengagement phenotype of Cep57-insufficient cells is recapitulated by Pcnt or Cdk5rap2 depletion. (A)** Analysis of knockdown efficiency of the indicated genes in MEFs using RT-qPCR. Two shRNAs were utilized for *Cep63*, *Cep152* and *Pcnt*. *Gapdh* was used as housekeeping gene. Expression was normalized to scrambled shRNA control. **(B)** Representative images of metaphase MEFs labeled for Cdk5rap2 to confirm knockdown. Metaphase cell with centrosome amplification is shown on right. **(C)** Representative images of interphase and metaphase MEFs labeled for Pcnt to confirm knockdown. **(D)** Representative images of interphase and metaphase MEFs labeled for Cep63 to confirm knockdown. **(E)** Representative images of interphase and metaphase MEFs labeled for Cep152 to confirm knockdown. **(F)** Western blot analysis showing knockdown efficiencies of Cep57 shRNAs. ShRNAs 2 and 3 were used for experiments. Analysis in **A** was performed on 3 independent wildtype MEF lines. Analyses in **B**-**E** were performed on at least 3 independent lines/genotype (20-25 cells/line). Data in **A** represent the mean ± SEM. Statistical significance was determined using a 2-tailed unpaired *t* test. $^*P < 0.05$, $^{**}P < 0.01$, $^{***}P < 0.001$Scale bars: 5 µm.

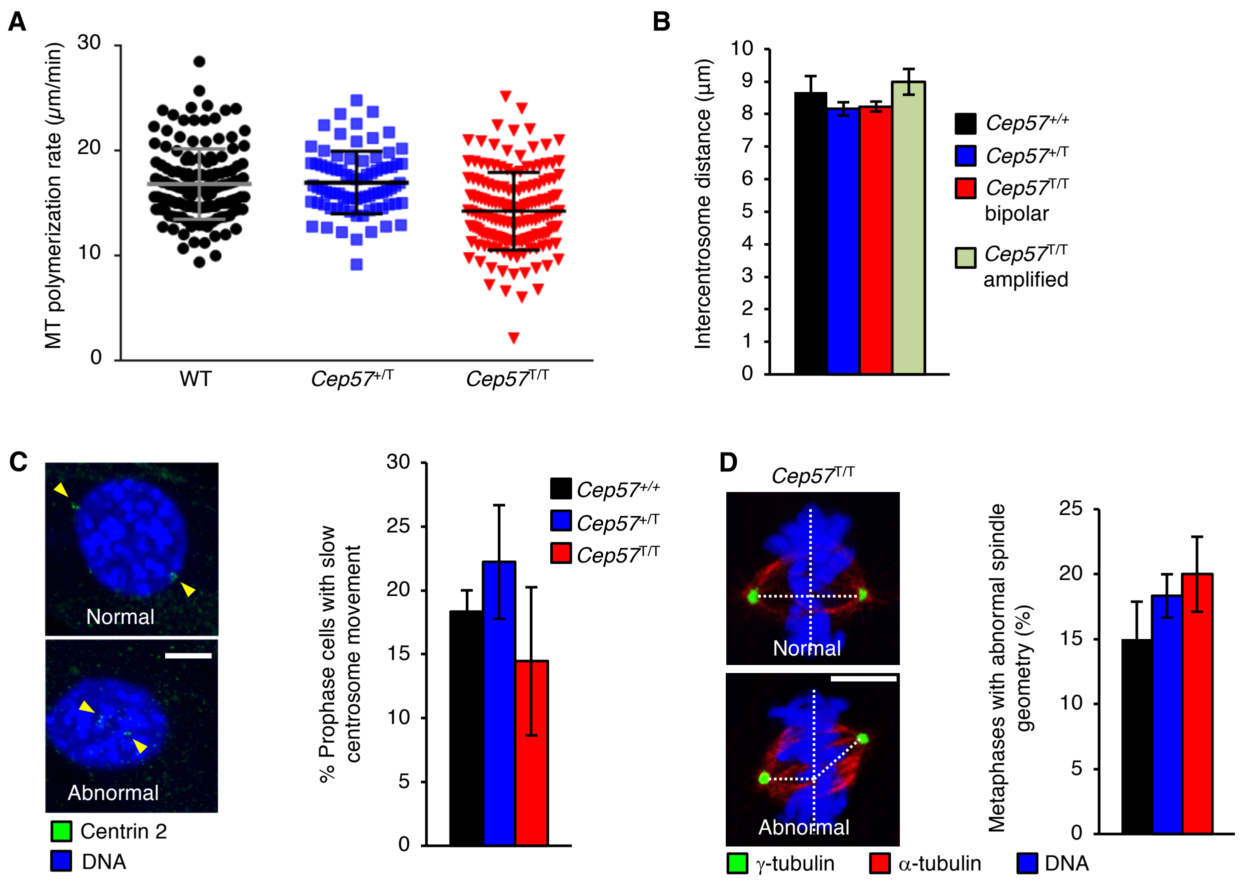
A
MT polymerization rate (μm/min)
30
20
10
0
WT
Cep57+/T
Cep57T/T
B
Intercentrosome distance (μm)
10
9
8
7
6
5
4
3
2
1
0
Cep57+/+
Cep57+/T
Cep57T/T
bipolar
Cep57T/T
amplified
C
Normal
Abnormal
Centrin 2
DNA
% Prophase cells with slow centrosome movement
30
25
20
15
10
5
0
Cep57+/+
Cep57+/T
Cep57T/T
D
Cep57T/T
Normal
Abnormal
γ-tubulin
α-tubulin
DNA
Metaphases with abnormal spindle geometry (%)
25
20
15
10
5
0

Supplemental Figure 6. Centrosome movement and spindle geometry are normal in Cep57 mutant MEFs lacking centrosome amplification. (A) Measurement of microtubule (+) end polymerization rate by EB3-GFP live cell imaging. **(B)** Measurement of inter-centrosomal distance in metaphase cells of indicated genotypes. Only cells with bipolar spindle orientation were analyzed. **(C)** Representative images of prophase cells with normal or reduced centrosome movement. Arrowheads indicate location of centrosomes. Quantification of cells with centrosomes separated by less than half of the longest diameter of a cell for indicated genotypes shown on right. **(D)** Representative images of metaphase cells with centrosomes aligned perpendicular to the axis of division or non-perpendicular to the axis. Quantification of cells that had imperfect alignment exceeding 5° (<85° or >95°) is shown on the right. Analyses in **B**-**D** were performed on at least 3 independent lines/genotype (20-25 cells/line). Analysis in **A** was performed on 2 lines/genotype, with 10 microtubule assembly rates measured for 10 cells per line. Data in **A**-**D** represent the mean ± SEM. Statistical significance was determined using a 2-tailed unpaired t test. Scale bars: 5 μm.

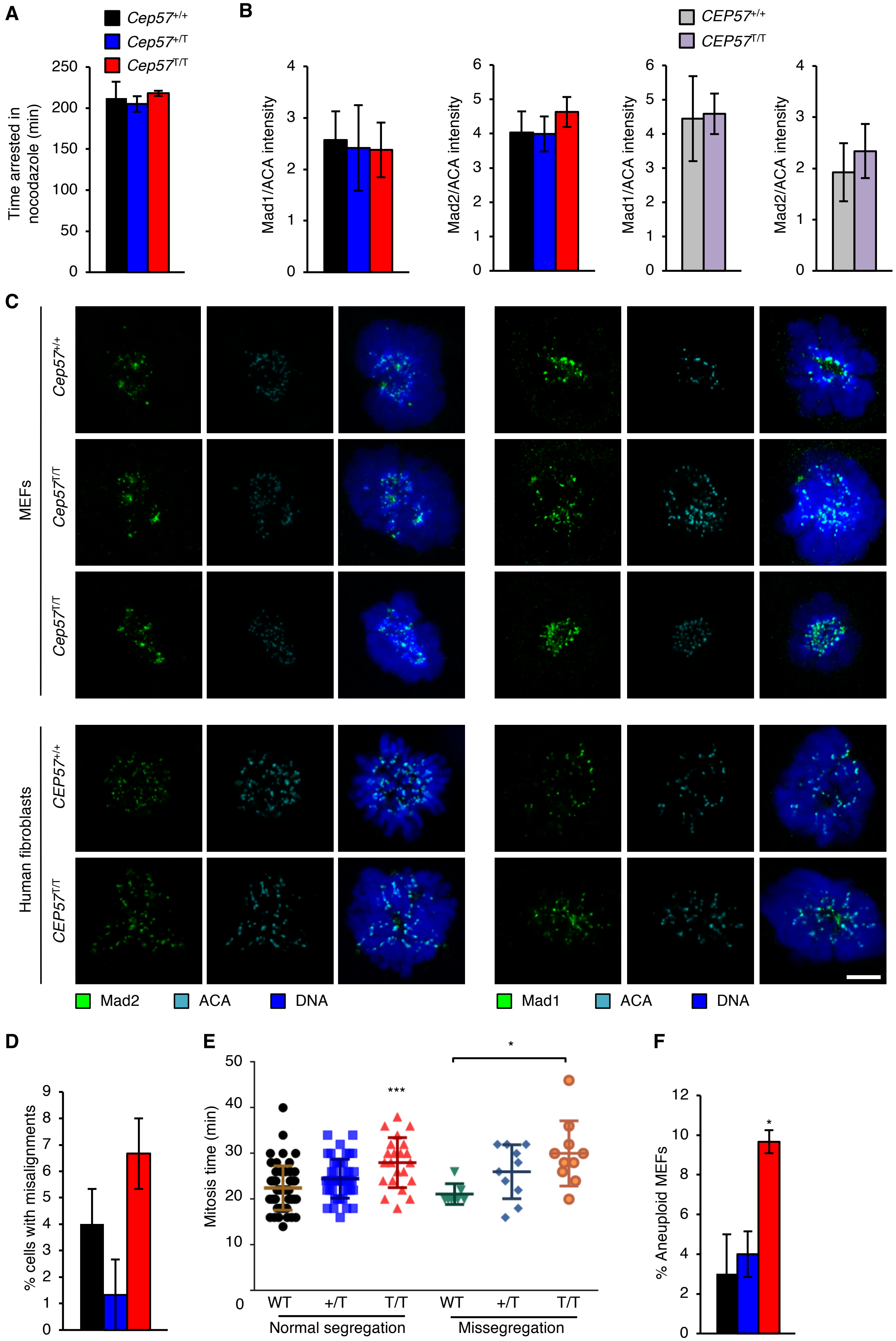
A
Cep57+/+
Cep57+/T
Cep57T/T
Time arrested in nocodazole (min)
B
CEP57+/+
CEP57T/T
Mad1/ACA intensity
Mad2/ACA intensity
Mad1/ACA intensity
Mad2/ACA intensity
C
MEFs
Cep57+/+
Cep57T/T
Cep57T/T
Human fibroblasts
CEP57+/+
CEP57T/T
Mad2
ACA
DNA
Mad1
ACA
DNA
D
% cells with misalignments
E
Mitosis time (min)
***
*
WT
+/T
T/T
WT
+/T
T/T
Normal segregation
Missegregation
F
% Aneuploid MEFs
*

Supplemental Figure 7. **Spindle assembly checkpoint activity appears normal in Cep57 insufficient MEFs.** **(A)** Time spent in mitotic arrest for H2B-mRFP expressing MEFs challenged with nocodazole, determined by live cell imaging. **(B)** Quantification of Mad1 and Mad2 kinetochore signals in MEFs and human fibroblasts of the indicated genotypes. Signals were normalized to those of anti-centromere antigen (ACA). **(C)** Representative images of prometaphase MEFs and human fibroblasts of the indicated genotypes stained for the indicated proteins. DNA was visualized with Hoechst. **(D)** Incidence of misaligned chromosomes in cells challenged with monastrol to induce monopolar spindles and then washed out to check error correction efficiency. **(E)** Time from nuclear envelope breakdown to anaphase onset in MEFs expressing H2B-mRFP. Cells with and without a chromosome missegregation event were analyzed separately. **(F)** Aneuploidy in interphase P0 MEFs analyzed by performing FISH using probes for chromosomes 4 and 7. Analyses in **A**-**F** were performed on at least 3 independent lines/genotype (20-25 cells/line). Analysis on human cells in **B** was repeated 3 times. Data in **A-B**, **D-F** represent the mean ± SEM. Statistical significance in **A-B**, **D** was determined using a 2-tailed unpaired *t* test, and in **E-F** by using a 1-way ANOVA, followed by Tukey's multiple comparisons test. *$P < 0.05$ and ***$P < 0.001$. Scale bars: 5 μm.

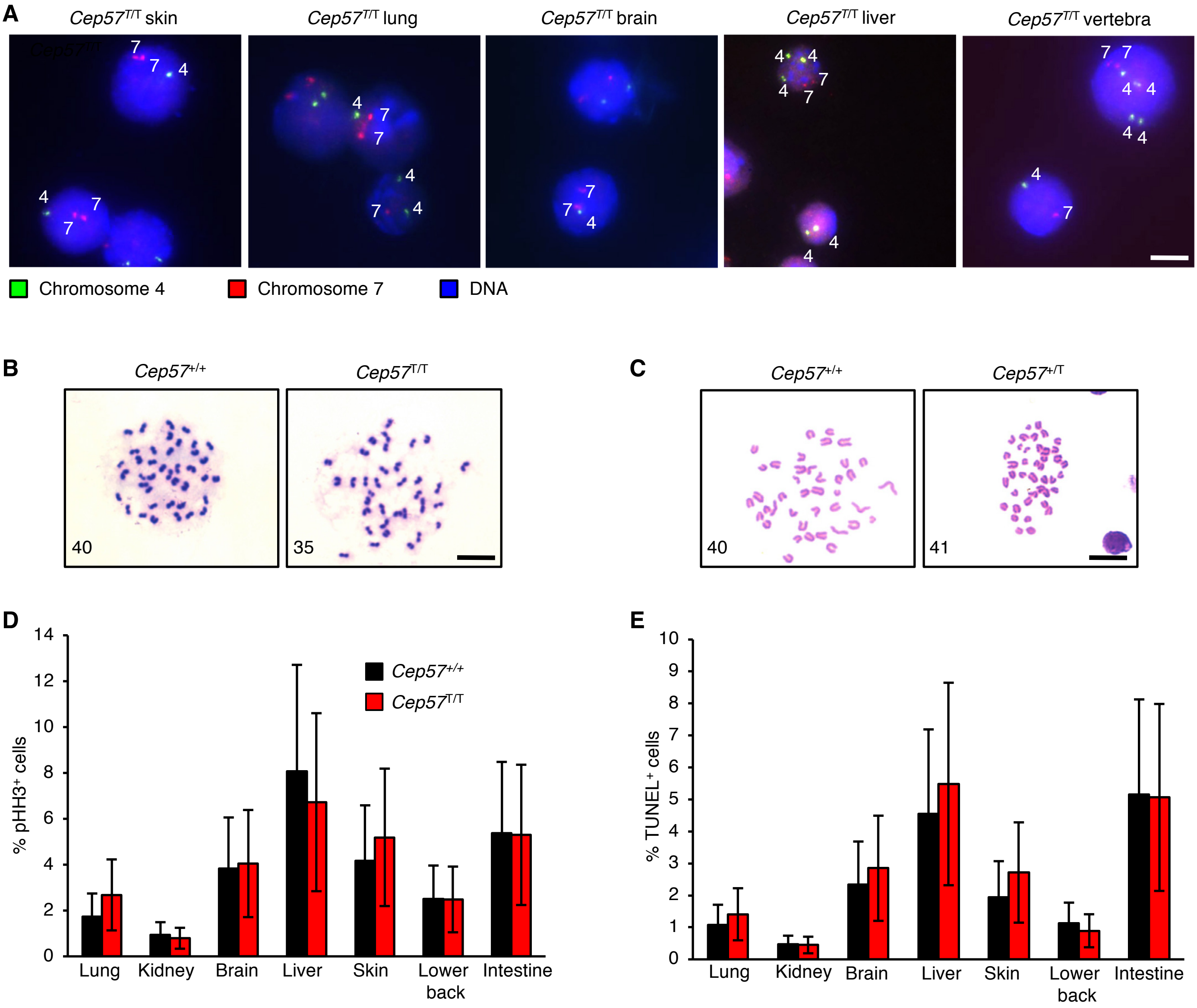
A
Cep57T/T skin
Cep57T/T lung
Cep57T/T brain
Cep57T/T liver
Cep57T/T vertebra
Chromosome 4
Chromosome 7
DNA
B
Cep57+/+
Cep57T/T
40
35
C
Cep57+/+
Cep57+/T
40
41
D
% pHH3+ cells
0
2
4
6
8
10
12
14
Cep57+/+
Cep57T/T
Lung
Kidney
Brain
Liver
Skin
Lower back
Intestine
E
% TUNEL+ cells
0
1
2
3
4
5
6
7
8
9
10
Lung
Kidney
Brain
Liver
Skin
Lower back
Intestine

Supplemental Figure 8. **MVA patient mimetic mice have widespread aneuploidy in vivo. (A)** Representative images of single cells prepared from indicated tissues of 1-day-old *Cep57*$^{T/T}$ mice stained for chromosomes 4 and 7 by FISH. **(B)** Representative images from karyotyping analysis performed on liver hematopoietic cells collected from 1-day-old mice of indicated genotypes. **(C)** Representative images from karyotyping analysis performed on splenocytes from 5 month old mice of indicated genotypes. **(D)** Percentage of cells positive for pHH3 in sagittal sections from 1-day-old animals. **(E)** Percentage of cells positive for TUNEL in sagittal sections from 1-day-old animals. At least 3 animals per genotype were used in **A**-**E**, with 100 cells per tissue quantified for each animal in **A**, **D** and **E**; 50 cells per animal in **B** and **C**. Data in **D**-**E** represent the mean ± SEM. Statistical significance was determined using a 2-tailed unpaired *t* test. Scale bar: 5 μm, 10 μm.

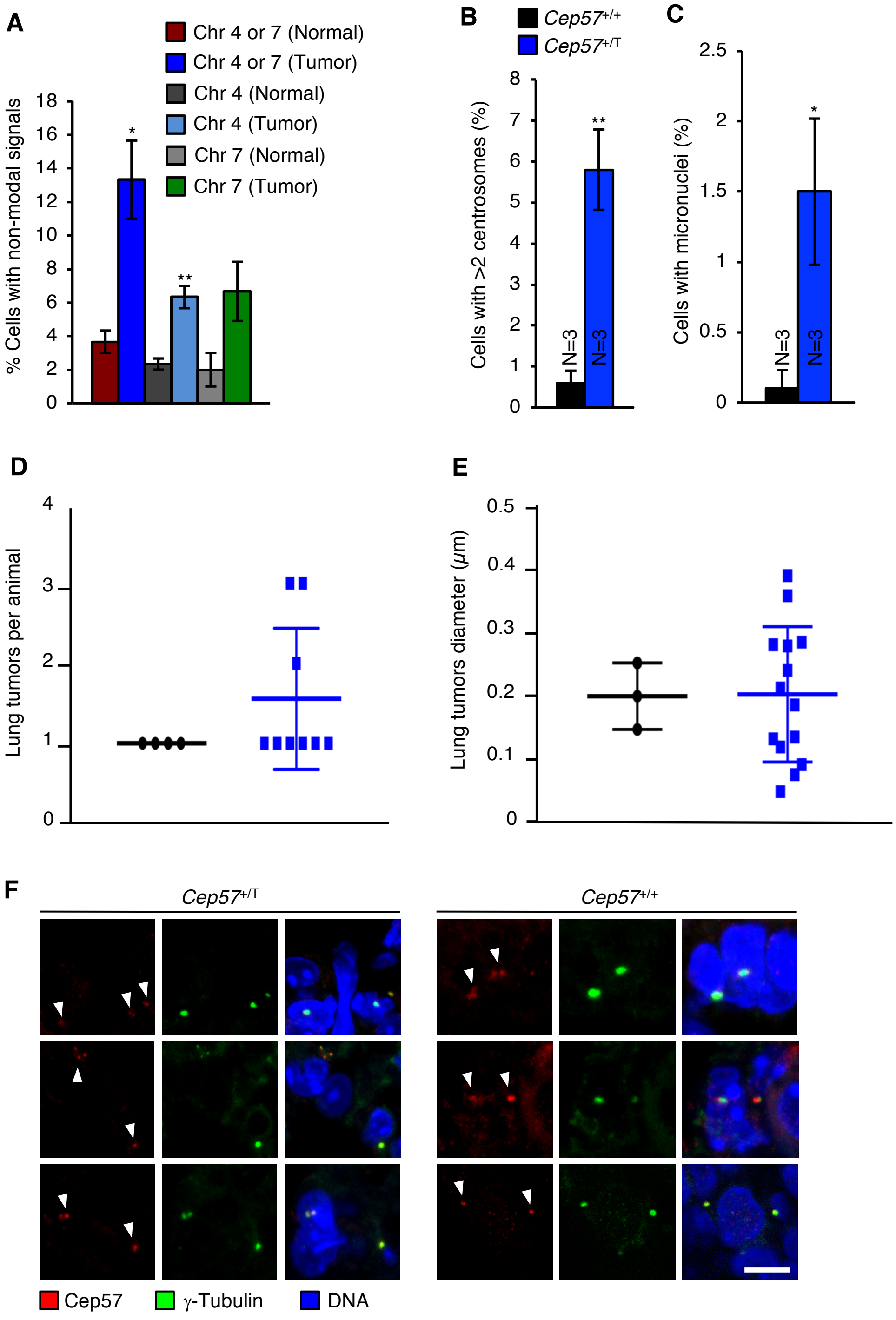

A
Chr 4 or 7 (Normal)
Chr 4 or 7 (Tumor)
Chr 4 (Normal)
Chr 4 (Tumor)
Chr 7 (Normal)
Chr 7 (Tumor)
% Cells with non-modal signals
B
Cep57+/+
Cep57+/T
Cells with >2 centrosomes (%)
N=3
N=3
C
Cells with micronuclei (%)
N=3
N=3
D
Lung tumors per animal
E
Lung tumors diameter (μm)
F
Cep57+/T
Cep57+/+
Cep57
γ-Tubulin
DNA

Supplemental Figure 9. Cep57-insufficient mice are cancer prone and form aneuploid tumors. **(A)** Percentage of single cells isolated from lung adenomas or flanking normal lung tissue from *Cep57*$^{+/T}$ mice with aneuploidy as determined by FISH using probes for chromosomes 4 and 7. **(B)** Percentage of cells with centrosome amplification in sections of lung adenomas and flanking normal tissue of 16-month-old *Cep57*$^{+/T}$ mice. Centrosomes were visualized using γ-tubulin immunostaining, and DNA with Hoechst. More than 50 cells were analyzed per tumor. **(C)** Quantification of micronuclei in the same tissue sections used in **B**. Micronuclei were visualized with Hoechst. More than 150 cells were counted per tumor. **(D)** Dot plot of lung tumor multiplicity in *Cep57*$^{+/+}$ and *Cep57*$^{+/T}$ mice treated with DMBA. **(E)** Dot plot of lung tumor size in *Cep57*$^{+/+}$ and *Cep57*$^{+/T}$ mice treated with DMBA. **(F)** High magnification images of spontaneous lung adenomas from *CEP57*$^{+/T}$ or wildtype mice after sectioning and immunolabeling for Cep57 (red) and γ-tubulin (green). DNA was stained with Hoechst. Arrowheads point to centrosomes. Three animals were used for the analysis in **A**-**C**. Samples sizes of 21 *Cep57*$^{+/T}$ and 20 *Cep57*$^{+/+}$ mice were used in **D** and **E**. Statistical significance in **A, D**, **E** was determined using a 2-tailed unpaired *t* test, and in **B** and **C** by 1-tailed, unpaired *t* test. *$P < 0.05$, **$P < 0.01$. Scale bar: 5 μm.

**Mitotic MEFs (n)**	**Mitotic figures inspected**	**Percent aneuploid figures (SD)**	**Karyotypes with indicated chromosome number**										
			**<35**	**35**	**36**	**37**	**38**	**39**	**40**	**41**	**42**	**43**	**>43**
Cep57 $^{+/+}$ (3)	150	9 (1.2)			1	1		10	137	1			
Cep57 $^{+/T}$ (3)	150	15 (1.2)**			1	2	2	7	130	3	3	2	
Cep57 $^{T/T}$ (3)	150	28 (2)***	1	3	1	3	4	11	108	15	1	3	
**Mitotic P1 liver cells (n)**	**Mitotic figures inspected**	**Percent aneuploid figures (SD)**	**Karyotypes with indicated chromosome number**										
			**<35**	**35**	**36**	**37**	**38**	**39**	**40**	**41**	**42**	**43**	**>43**
Cep57 $^{+/+}$ (3)	150	1.3 (1.2)				1		1	148				
Cep57 $^{+/T}$ (3)	146	8.8 (2.07)**	2		2		1	6	133	2			
Cep57 $^{T/T}$ (3)	147	29.9 (7.3)**		3	5	4	5	12	103	7	3	1	4
**Mitotic 5-month splenocytes (n)**	**Mitotic figures inspected**	**Percent aneuploid figures (SD)**	**Karyotypes with indicated chromosome number**										
			**<35**	**35**	**36**	**37**	**38**	**39**	**40**	**41**	**42**	**43**	**>43**
Cep57 $^{+/+}$ (3)	150	0.7 (1.2)					1		149				
Cep57 $^{+/T}$ (3)	150	21.3 (4.2)**				2	13	11	118	6			
**Mitotic 5-month splenocytes (n)**	**Mitotic figures inspected**	**Percent aneuploid figures (SD)**	**Karyotypes with indicated chromosome number**										
			**<35**	**35**	**36**	**37**	**38**	**39**	**40**	**41**	**42**	**43**	**>43**
Cep57 $^{+/+}$ (3)	150	0.7 (1.2)			1				149				
Cep57 $^{+/-}$ (3)	150	17 (3.1)***	1				3	12	124	5	4	1	

Supplemental Table 1. *Cep57* insufficiency causes aueuploidy. Karyotype analysis of numerical chromosomal abnormalities in either MEFs or liver hematopoetic cells harvested from 1-day-old mice or splenocytes from 5-month-old mice of indicated genotypes. Data are mean ± SD Statistical significance was evaluated by 1-way ANOVA for tissues and 2-tailed unpaired *t* test.for MEFs. *$P < 0.05$; **$P < 0.01$; ***$P < 0.001$.

Tissue/Cell type (n)	Genotype	Percentage of aneuploidy (SD)		
		Chr. 4	Chr. 7	Chr. 4/7
**MEFs, P0 (3))**	*Cep57*$^{+/+}$	2.3 (0.3)	1.7 (0.3)	3 (0.6)
	Cep57$^{+/T}$	3 (1.7)	1.7 (0.3)	4 (1.2)
	Cep57$^{T/T}$	6.3 (1.2)*	5.7 (1.2)*	9.7 (2)*
**Skin**	*Cep57*$^{+/+}$	2 (0.6)	0.7 (0.3)	2.6 (0.9)
	Cep57$^{+/T}$	1.7 (0.9)	0.7 (0.7)	2.3 (1.5)
	Cep57$^{T/T}$	3 (0.6)	5 (1.2)*	6.7 (0.9)*
**Lung**	*Cep57*$^{+/+}$	1 (0.6)	1 (0.6)	1.3 (0.3)
	Cep57$^{+/T}$	1.3 (0.7)	2 (0.6)	3 (1)
	Cep57$^{T/T}$	4.3 (1.5)	3.7 (1.3)	6.3 (1.7)*
**Brain**	*Cep57*$^{+/+}$	1 (0.6)	0 (0)	1 (0.6)
	Cep57$^{+/T}$	1.3 (0.7)	0.3 (0.3)	1.3 (0.7)
	Cep57$^{T/T}$	3 (1.2)	5 (1.2)*	5.3 (0.9)*
**Liver**	*Cep57*$^{+/+}$	2.3 (1.3)	1.3 (0.9)	2.3 (1.3)
	Cep57$^{+/T}$	1.3 (0.7)	1.3 (0.3)	2.3 (0.9)
	Cep57$^{T/T}$	3.7 (0.3)	4.3 (0.9)	7.7 (0.7)*
**Vertebra**	*Cep57*$^{+/+}$	0 (0)	0.6 (0.3)	0.7 (0.3)
	Cep57$^{+/T}$	2.3 (1.2)	2.6 (1.5)	4 (2)
	Cep57$^{T/T}$	1.3 (0.7)	2.3 (0.9)	3 (0.6)*

Supplemental Table 2. Interphase FISH in *Cep57* insufficient models show aneuploidy in a broad spectrum of tissues. Interphase FISH analysis on passage 0 MEFs or tissues of 1-day-old mice of indicated genotypes using probes for chromosomes 4 or 7. n = 3 per genotype, 100 cells per sample. Data are mean ± SD. Statistical significance was evaluated by 1-way ANOVA for tissues and 2-tailed unpaired t test for MEFs. *$P < 0.05$.
